# Supplementary material for: The proline-rich antimicrobial peptide Api137 disrupts large ribosomal subunit assembly and induces misfolding
Source: Nat Commun. 2025 Jan 10;16:567. doi: 10.1038/s41467-025-55836-8 (PMC11723945; doi:10.1038/s41467-025-55836-8)
Supplement: Supplementary file 1 — Supplementary Info [file 41467_2025_55836_MOESM1_ESM.pdf]

Supplementary Information

The proline-rich antimicrobial peptide Api137 disrupts large ribosomal subunit assembly and induces misfolding

Simon M. Lauer[1,2,#], Jakob Gasse[3,4,#], Andor Krizsan[3,4], Maren Reepmeyer[3,4], Thiemo Sprink[5,6], Rainer Nikolay[1,7✉], Christian M. T. Spahn[1✉], and Ralf Hoffmann[3,4✉].

# authors contributed equally and thus both have the right to use their names on the first place

✉ Corresponding authors: Dr. Rainer Nikolay (nikolay@molgen.mpg.de), Prof. Christian M. T. Spahn (christian.spahn@charite.de) and Prof. Ralf Hoffmann (bioanaly@rz.uni-leipzig.de)

Affiliation:

[1]Institut für Medizinische Physik und Biophysik, Charité - Universitätsmedizin Berlin, corporate member of Freie Universität Berlin and Humboldt Universität zu Berlin, Berlin, Germany.

[2]Humboldt-Universität zu Berlin, Institut für Biologie, 10099 Berlin, Germany

[3]Institute of Bioanalytical Chemistry, Faculty of Chemistry and Mineralogy, Universität Leipzig, Leipzig, Germany.

[4]Center for Biotechnology and Biomedicine, Universität Leipzig, Leipzig, Germany.

[5]Core Facility for Cryo-Electron Microscopy, Charité - Universitätsmedizin Berlin, Berlin, Germany

[6]Cryo-EM Facility, Max Delbrück Center for Molecular Medicine in the Helmholtz Association, Berlin, Germany

[7]Department of Genome Regulation, Max Planck Institute for Molecular Genetics, Berlin, Germany.

Table of contents

Supplementary Fig. 1: Ribosome profile analysis of *E. coli* RN31 in the presence of Onc112, Api137, Api88, Chloramphenicol and Erythromycin 3

Supplementary Fig. 2: Initial class identification and alignment 4

Supplementary Fig. 3: Identification of subclasses in Api137 treated samples 6

Supplementary Fig. 4: Sorting scheme of control data set 8

Supplementary Fig. 5: Cryo-EM maps, models, RNA composition, local resolutions and Model-map fits identified precursors 9

Supplementary Fig. 6: Global FSC curves and angular distribution plots of Api137-precursor maps 12

Supplementary Fig. 7: Comparison with *in vitro* reconstituted pre-50S states 14

Supplementary Fig. 8: All late precursors show incorporated uL22 15

Supplementary Fig. 9: Structures of pre-50S intermediates suggesting delayed and rerouted assembly pathways upon Api137 treatment 16

Supplementary Fig. 10: Sorting scheme of the particles from Api137-treated cells in the presence of 30 µM Api137.. 18

Supplementary Fig. 11: Cryo-EM maps, models, local and global resolutions and angular distributions of pooled refinements of Api137-precursors in the presence of 30 µM Api137.. 22

Supplementary Fig. 12: Absence and presence of Api137 at the PET binding site 24

Supplementary Fig. 13: Api137 binding to pre-50S precursors and 50S at the PET exit binding site… 26

Supplementary Tab. 1: Cryo-EM data collection, refinement, and validation statistics for the control data set … 28

Supplementary Tab. 2: Cryo-EM data collection, refinement, and validation statistics for the data set from Api137-treated cells ….. 29

Supplementary Tab. 3: Cryo-EM data collection, refinement, and validation statistics for the data set from Api137-treated cells supplemented with Api137… 31

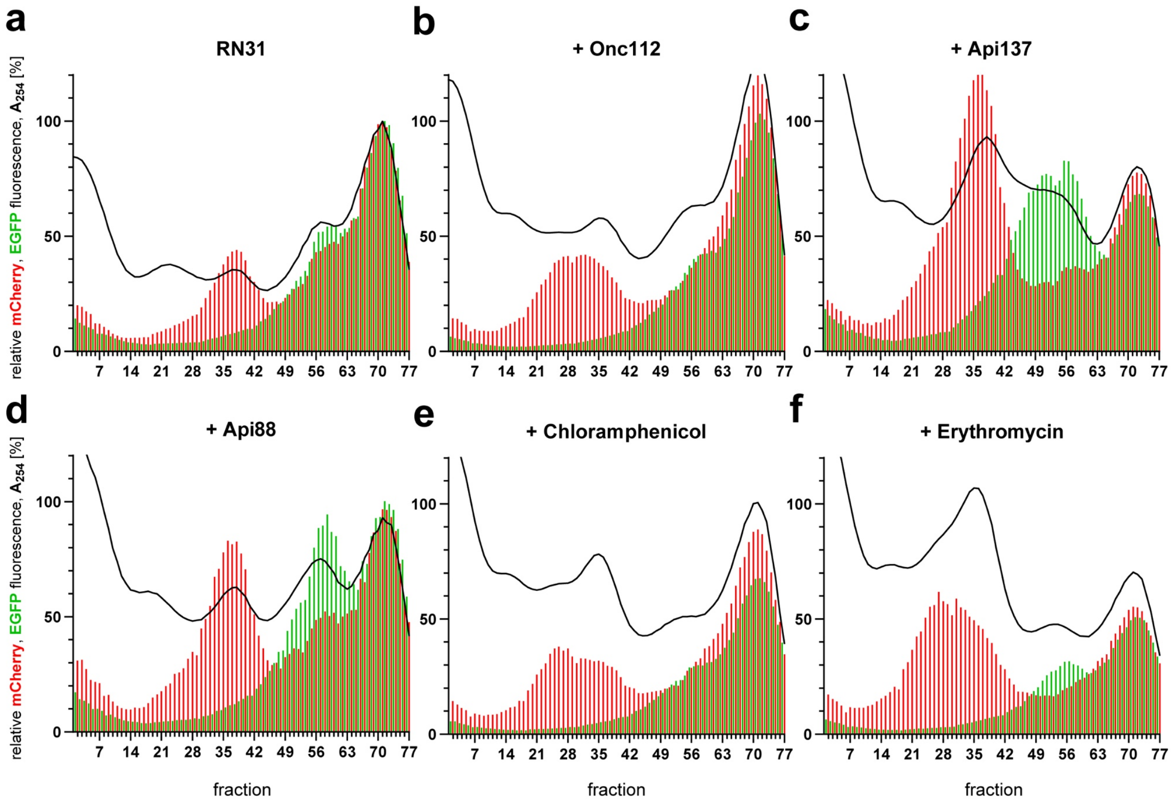

Supplementary Fig. 1 | Ribosome profile analysis of *E. coli* RN31 in the presence of Onc112, Api137, Api88, Chloramphenicol and Erythromycin. a - f All absorbance and fluorescence values were normalized to the mean value of the corresponding maximum peak intensity of the 70S fraction in the control. OD_{254} (black curve) and EGFP and mCherry fluorescence values (green and red bars, respectively) obtained for **a** *E. coli* RN31 grown in culture medium (n = 10) and incubated with **b** Onc112 (8 µg/mL, n = 6), **c** Api137 (8 µg/mL, n = 10), **d** Api88 (8 µg/mL, n = 2), **e** chloramphenicol (8 µg/mL, n = 6) or **f** erythromycin (128 µg/mL, n = 6) along a sucrose gradient from 5 to 25%.

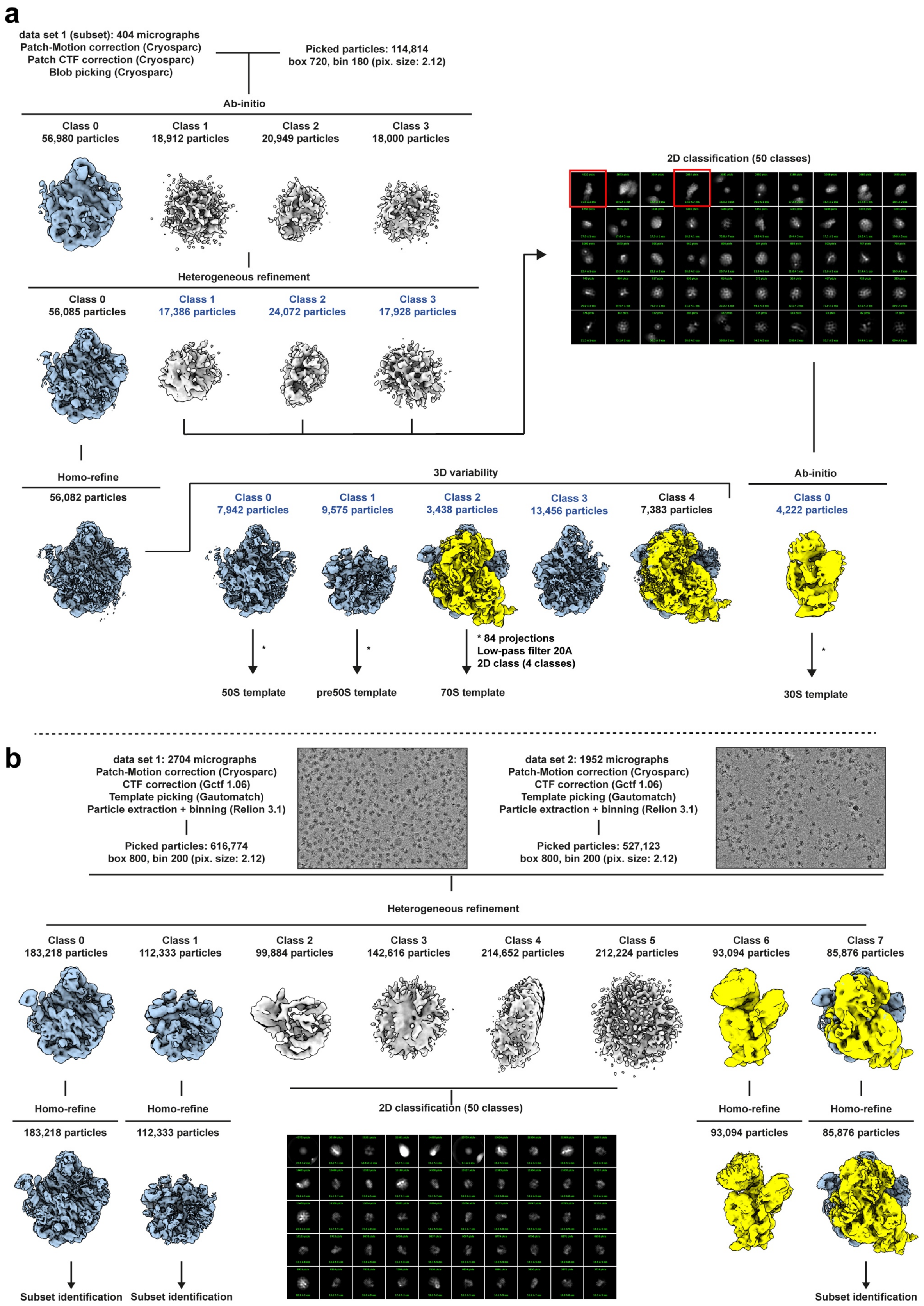

Supplementary Fig. 2 | Initial class identification and alignment. a A subset of data set 1 was pre-processed in CryoSPARC using patch-motion correction (standard parameters) and patch-CTF correction (resolution range: 12 Å - 4 Å). Particles were picked using a blob picker with a particle

diameter ranging from 140 to 220 Å. Particles were extracted with a box size of 720 Å and binned on-the-fly to 180 Å for an initial pixel size of 2.12 Å. Particles were subjected to one round of multi-class ab-initio reconstruction (class similarity = 0) for an initial consensus map, followed by heterogeneous refinement to discard non-ribosomal particles. A consensus refinement was performed on the ribosomal map, followed by a 3D variability analysis. The variability analysis revealed the presence of 70S, 50S and pre-50S classes. 30S was identified using a 2D classification, followed by selection of respective classes and template generation using ab-initio reconstruction. To improve particle picking, 84 equally spaced projections from each 3D volume (70S, 30S, 50S, pre-50S) were generated in CryoSPARC and filtered to 20 Å, followed by Xmipp3 2D classification (four classes for each set of 84 projections) to generate generalized picking templates. 2D classes were added to one stack for a final set of 16 picking templates. Particles were picked again, now using both datasets and the previously generated templates in Gautomatch (developed by K. Zhang). Particle images were extracted and normalized, using Relion 3.1 [45] with a box size of 800 and Fourier cropped to 200 for sorting. **b** Newly extracted particles from both data sets were reassigned to the previously identified 70S, 50S, 30S and pre-50S states and non-ribosomal classes, followed by a homogenous refinement. Particles to be discarded were double-checked for remaining ribosomal particles using a 2D classification.

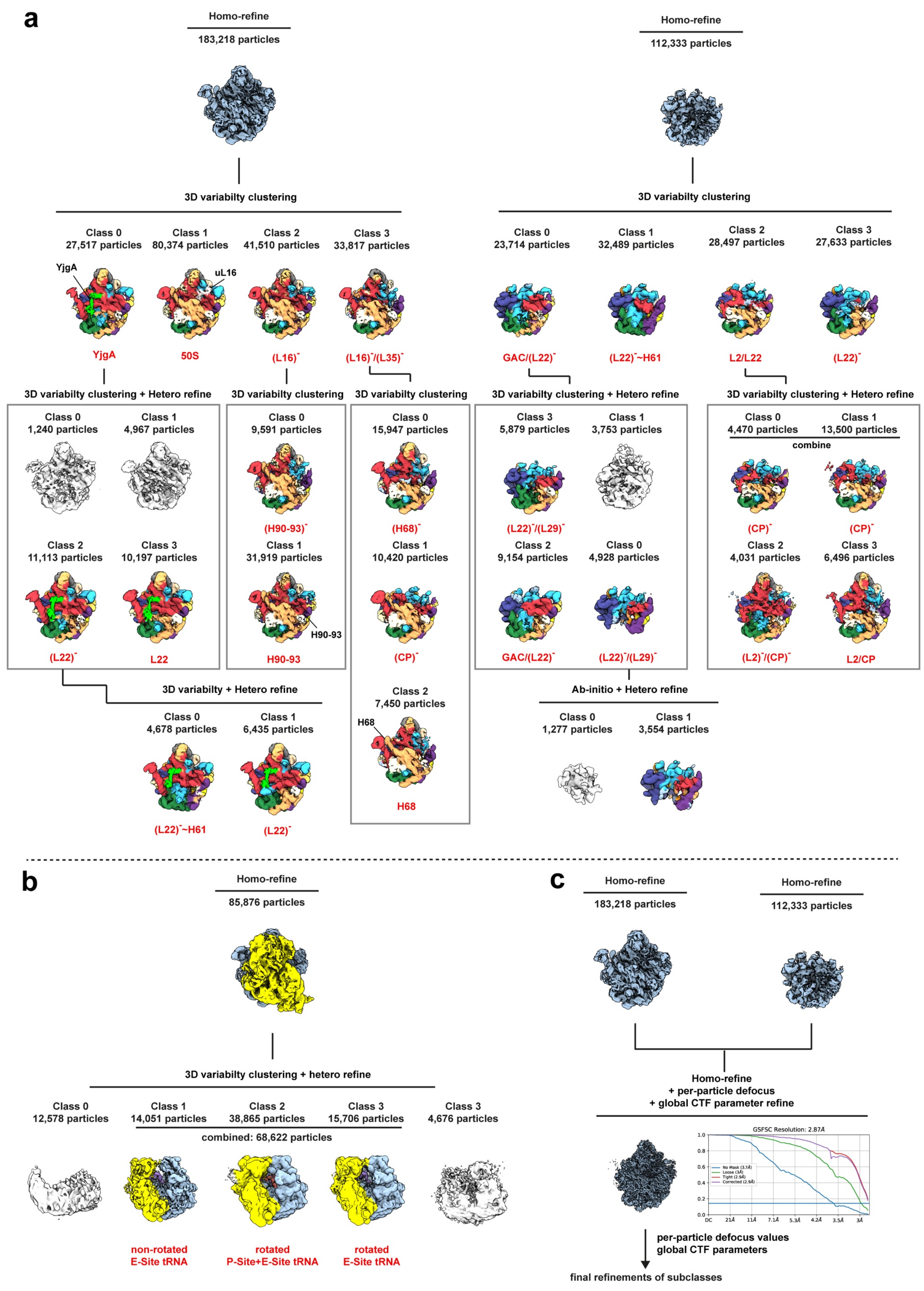

Supplementary Fig. 3 | Identification of subclasses in Api137 treated samples. a 50S and pre-50S states were subjected to multiple rounds of 3D variability analysis, until there was no more separation of distinct states. When denoted, clusters from 3D variability analyses were used in a Heterogenous

refinement for an additional re-assignment step. **b** Principle 70S states were identified using 3D variability analysis, followed by an additional Heterogenous refinement. Non-defined classes of 70S, 50S and pre-50S states were discarded. **c** To improve per-particle CTF values, 50S and pre-50S states were aligned to a single volume using an on-the-fly global and local CTF correction. Final refinements of distinct substrates were performed using optimized CTF values from this consensus refinement.

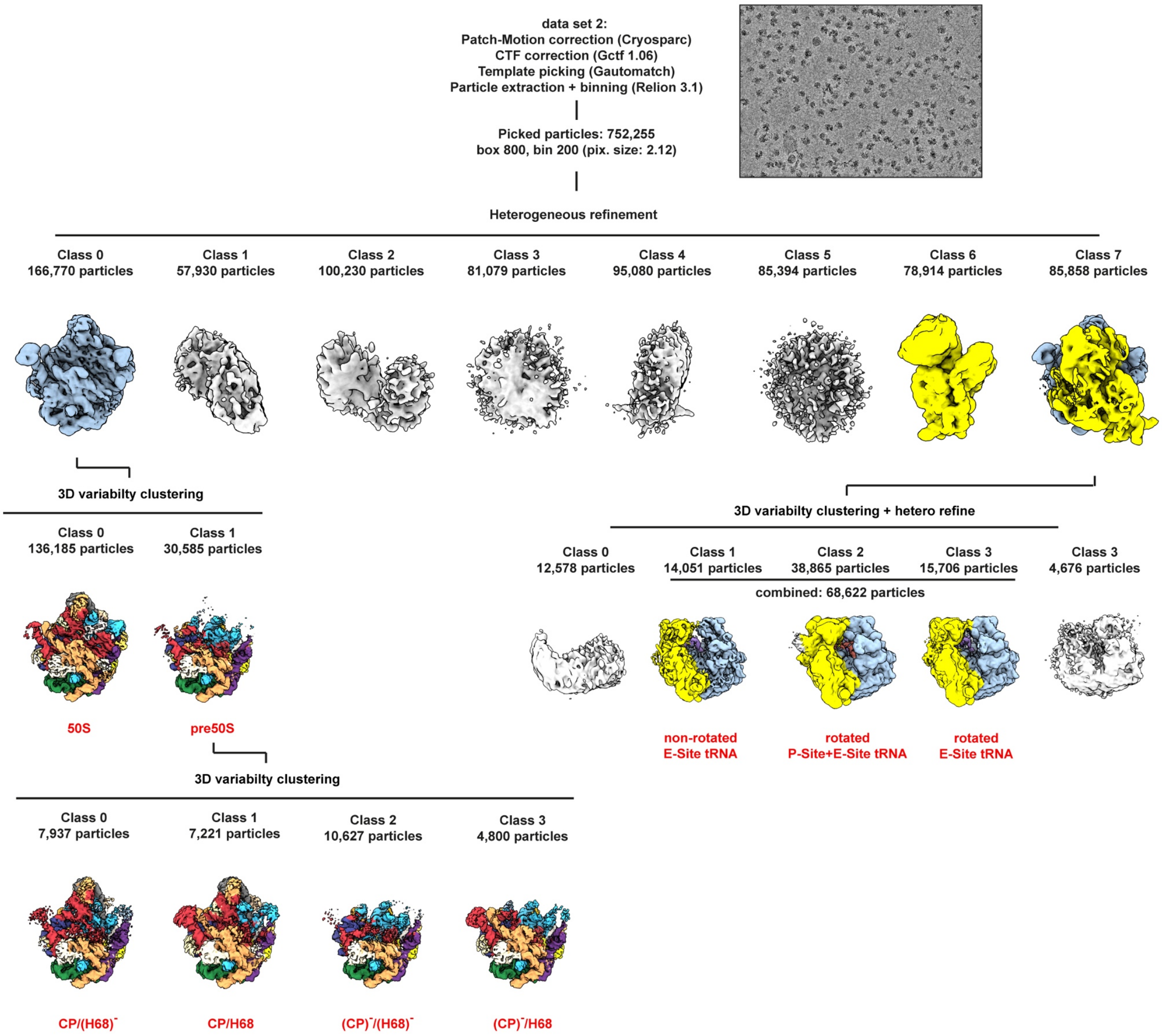

Supplementary Fig. 4 | Sorting scheme of control data set. The control data set was pre-processed in CryoSPARC using patch-motion correction (standard parameters) and patch-CTF correction (resolution range: 12 Å – 4 Å). Particles were picked using a blob picker with a particle diameter ranging from 140 to 220 Å. Particles were extracted with a box size of 720 Å and binned on-the-fly to 180 Å for an initial pixel size of 2.12 Å. Particles were subsequently assigned to the principal and non-ribosomal classes, identified in the Api137 data set. Particles corresponding to 50S, 30S, and 70S classes were refined once and subjected to 3D variability analysis until no more separation was observed.

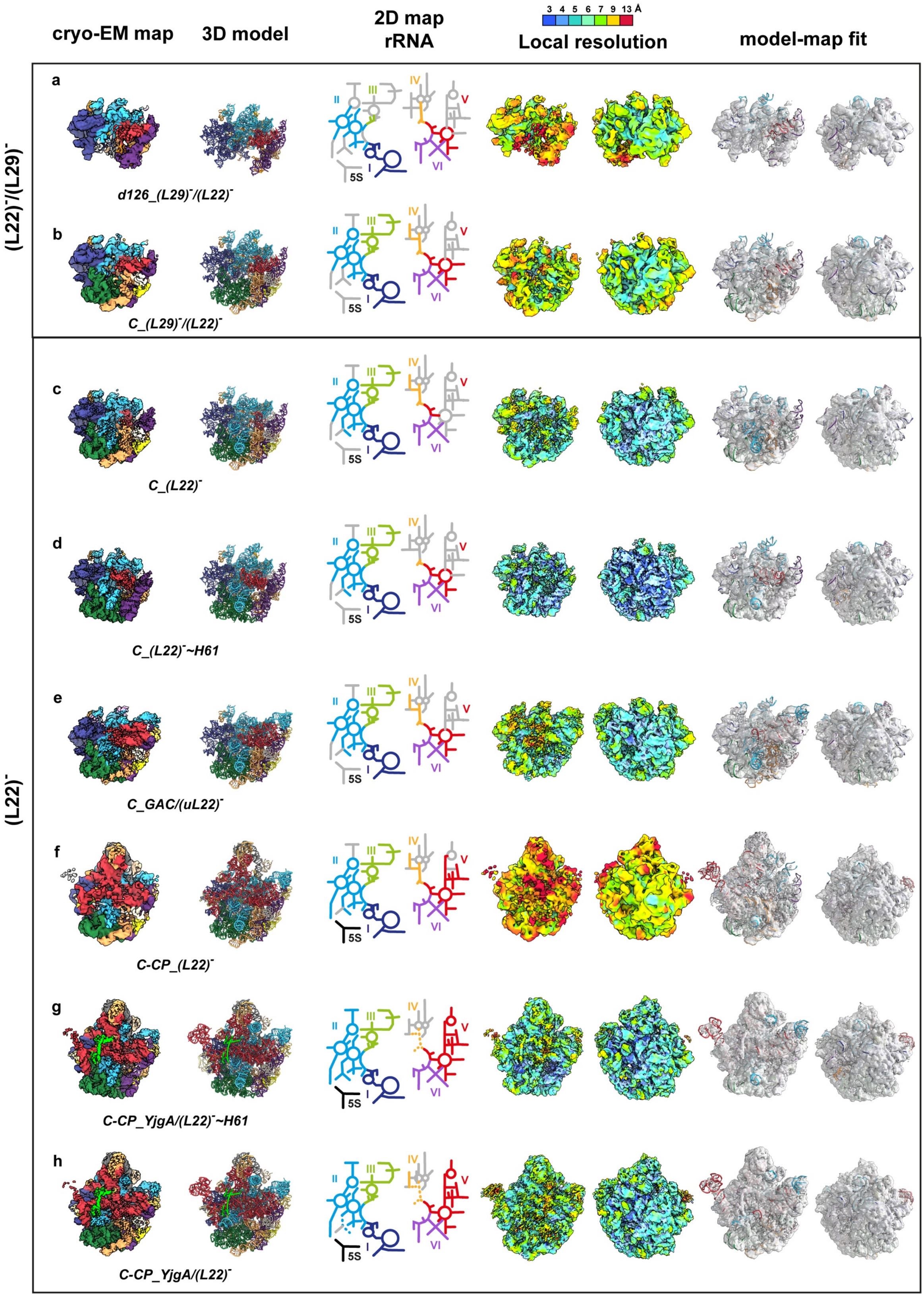
cryo-EM map
3D model
2D map rRNA
3 4 5 6 7 9 13 Å
Local resolution
model-map fit
(L22)⁻/(L29)⁻
a
d126_(L29)⁻/(L22)⁻
b
C_(L29)⁻/(L22)⁻
(L22)⁻
c
C_(L22)⁻
d
C_(L22)⁻~H61
e
C_GAC/(uL22)⁻
f
C-CP_(L22)⁻
g
C-CP_YjgA/(L22)⁻~H61
h
C-CP_YjgA/(L22)⁻
I
II
III
IV
V
VI
5S

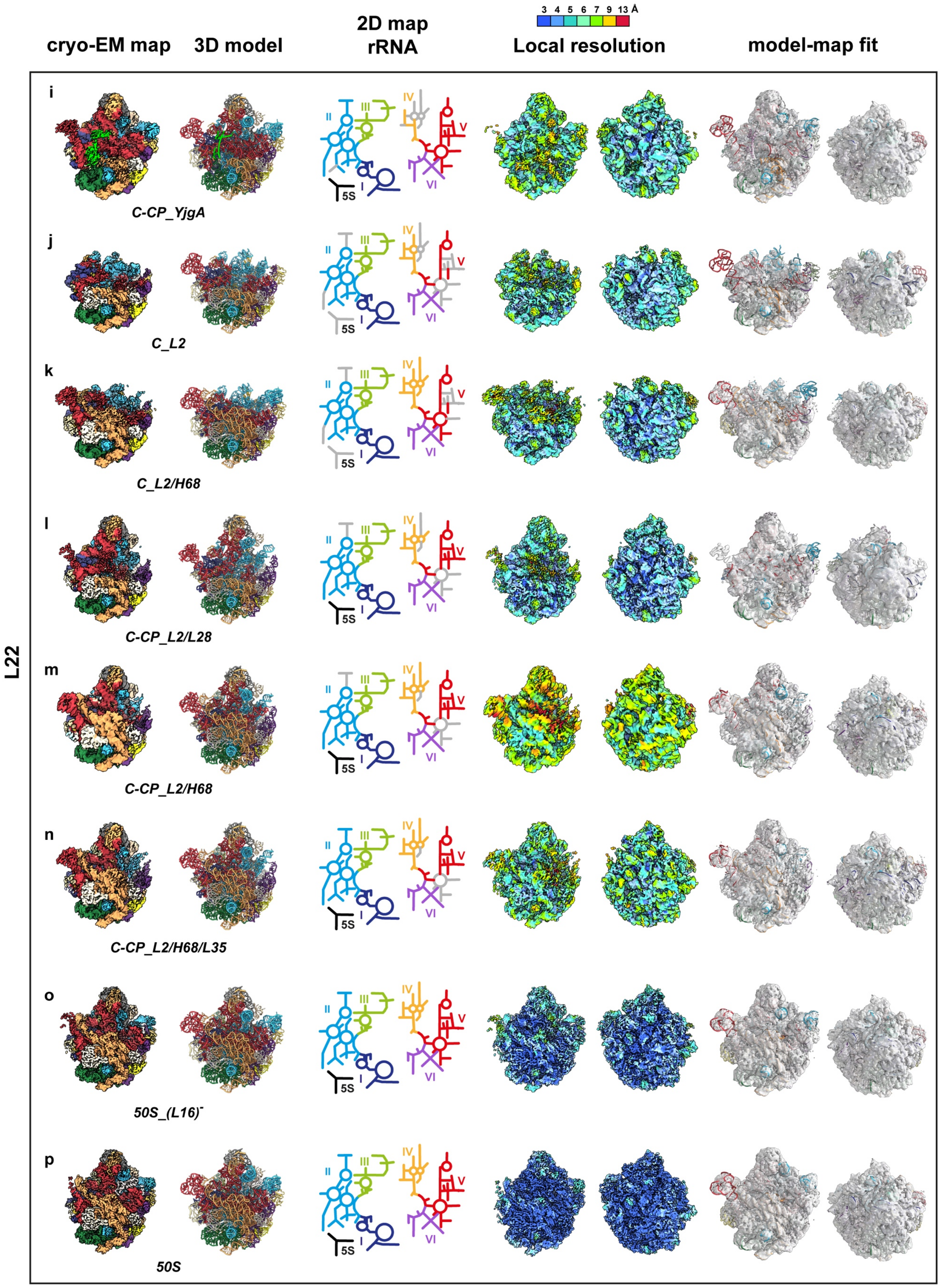

Supplementary Fig. 5 | cryo-EM maps, models, RNA composition, local resolutions and model-map fits of identified precursors. a-p States were lined up by their degree of structural completeness. Cryo-EM maps with rRNA and L-proteins color-coded according to the six architectural domains of the 23S rRNA they are part of. 3D PDB models, and 2D maps with the six rRNA domains color-coded. Colored regions indicate stably formed segments, for which cryo-EM densities were obtained.

Additionally, local resolutions (crown and back view) are shown for each state. 3D PDB models color-coded according to the six architectural domains of the 23S rRNA. Model-map fits show cryo-EM maps and models of all states a in crown and back view. Cryo-EM maps for this analysis were derived by subjecting final states to an additional ab-initio reconstruction, followed by Homogenous refinement, shown at a threshold of 0.33. Remaining heterogeneity in parts of the individual states is indicated in **bold**: **a** *d126_(L29)*$^{-}$*/(L22)*$^{-}$: (**uL6**, **bL30**), **b** *C_(L29)*$^{-}$*/(L22)*$^{-}$: (**uL6**, **bL30**), **c** *C_(L22)*$^{-}$: (**H34**), **d** *C_(L22)*$^{-}$*~H61*: (**bL30**), **e** *C_GAC(L22)*$^{-}$: (**bL30, H34, H63**), **f** *C-CP_YjgA_(L22)*$^{-}$*~H61*, **g** *C-CP_YjgA_(L22)*$^{-}$, **h** *C-CP_YjgA*: **YjgA**, **i** *C_L2*: (**uL6, uL22, bL30**), **j** *C_H68*: (**uL6, bL30, H90-93**), **k** *C-CP_L2/L28*, **l** *C-CP_(L22)*$^{-}$: (**uL22**), **m** *C-CP_H68*: (**H90-93**), **n** *C-CP_H68_L35*: (**H90-93**), **o** *50S_(L16)*$^{-}$, **p** *50S.*

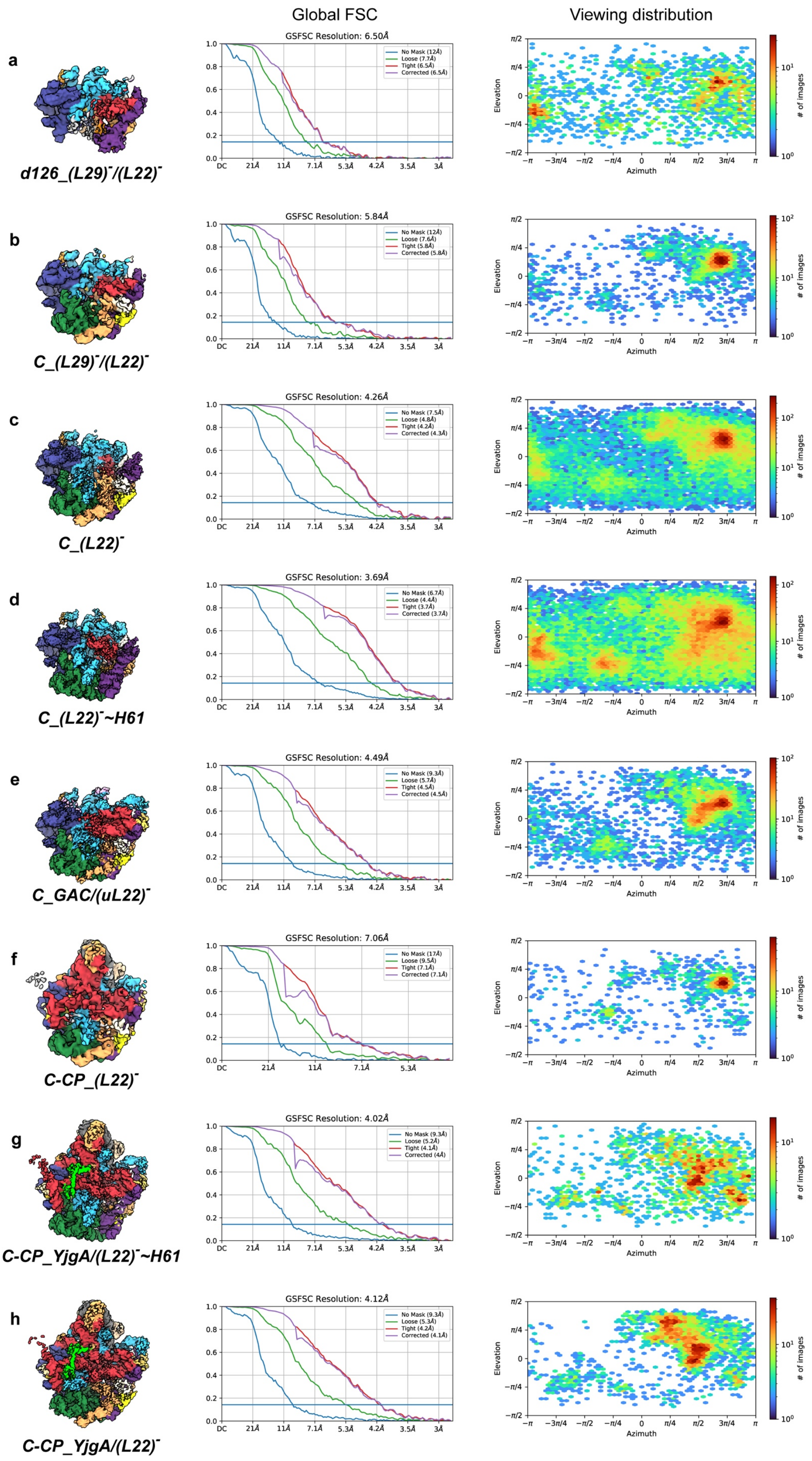

Global FSC
Viewing distribution
a
d126_(L29)-/(L22)-
GSFSC Resolution: 6.50Å
b
C_(L29)-/(L22)-
GSFSC Resolution: 5.84Å
c
C_(L22)-
GSFSC Resolution: 4.26Å
d
C_(L22)-~H61
GSFSC Resolution: 3.69Å
e
C_GAC/(uL22)-
GSFSC Resolution: 4.49Å
f
C-CP_(L22)-
GSFSC Resolution: 7.06Å
g
C-CP_YjgA/(L22)-~H61
GSFSC Resolution: 4.02Å
h
C-CP_YjgA/(L22)-
GSFSC Resolution: 4.12Å
Elevation
Azimuth
# of images

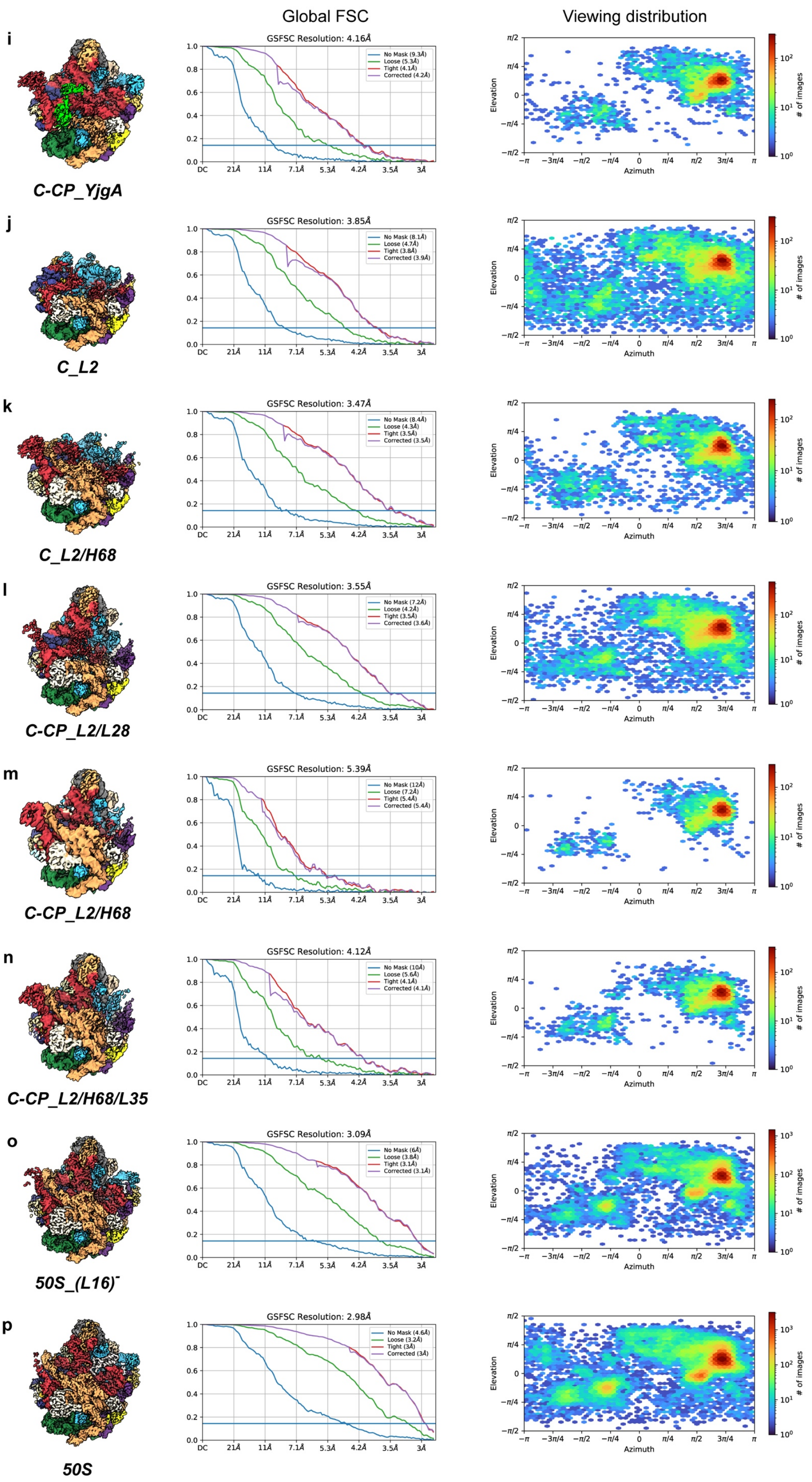

Supplementary Fig. 6 | Global FSC curves and angular distribution of pre-50S precursor maps. **a-p** Cryo-EM maps of all states are shown. For each state, the global Fourier-shell correlation (FSC) curves with estimated global resolutions at the 0.143 cut-off, as well as the angular distribution plots of the final reconstructions, are provided.

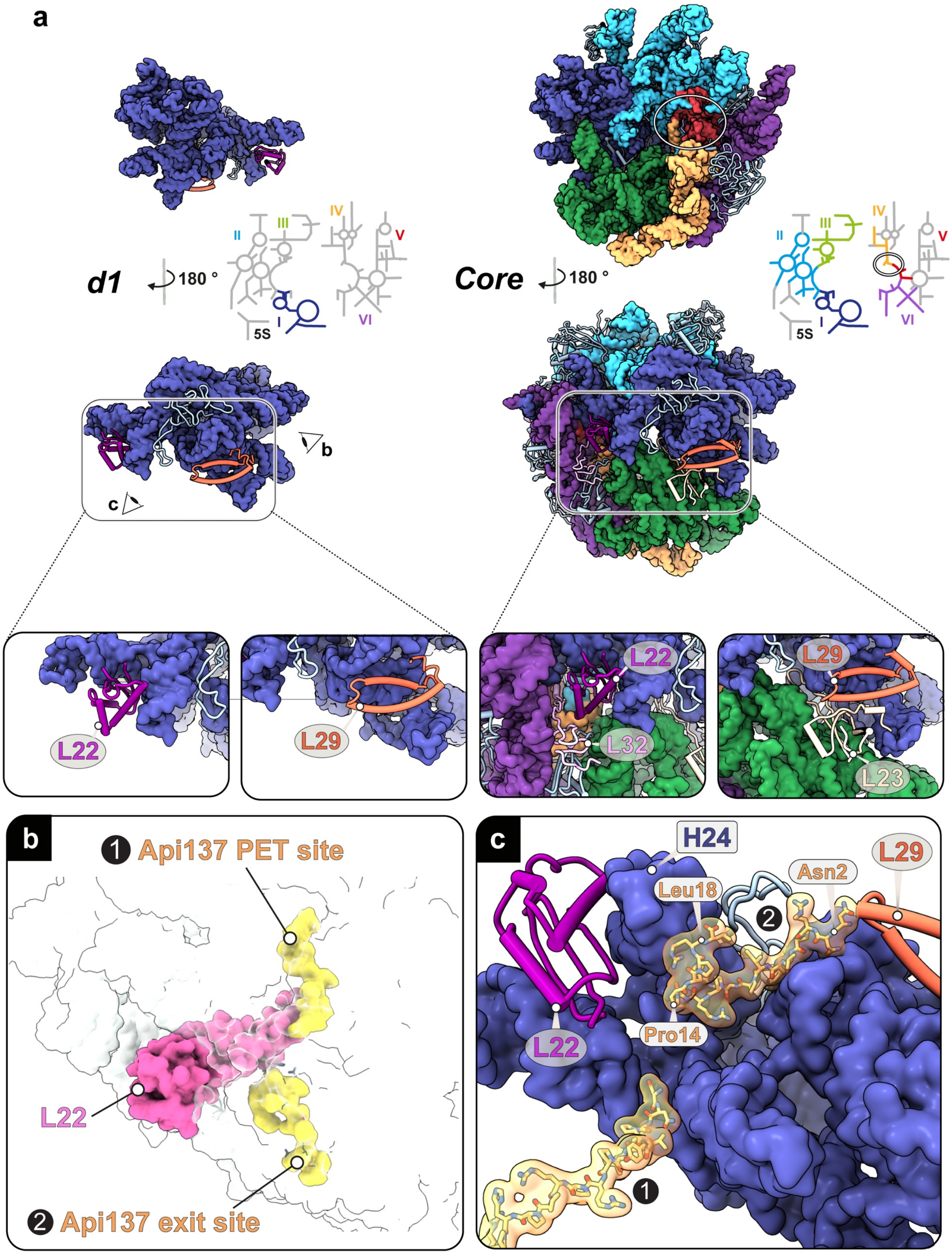

Supplementary Fig. 7 | Comparison with *in vitro* reconstituted pre-50S states. a Previously identified hallmark assembly states *d1* and *C* (core) are shown. Structures are shown as a surface model at 5 Å resolution and color-coded according to the 23S rRNA domain architecture. 2D plots show structured regions of the entire subunit. Enlargements show the positions of uL22, uL29, bL32, and bL23. Viewing angles in **b** and **c** are indicated using schematic eyes. **b** Previously identified positions of the PET and PET exit binding sites of Api137 (yellow) on the 50S subunit are shown relative to uL22 (pink). uL22 and Ap137 are shown as a surface models. **c** Atomic models and simulated densities at 4

Å resolution of both Api137 binding sites on the 50S subunit are superimposed on the assembly state *d1*. Maps and models in **a** and **b** taken from Lauer et al., 2024 (PDB: 8RPY).

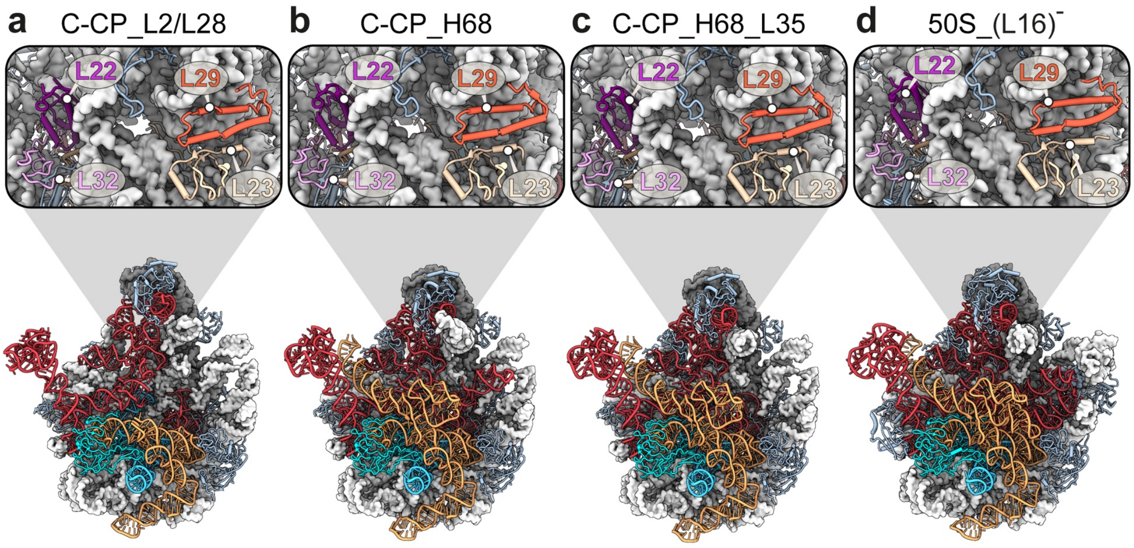

Supplementary Fig. 8 | All late precursors show incorporated uL22. Late assembly states identified in Api137 *ex vivo* precursors. Helix H34 and helices in domains IV and V are shown as ribbons, the remaining subunit is shown as a surface model at 5 Å resolution.

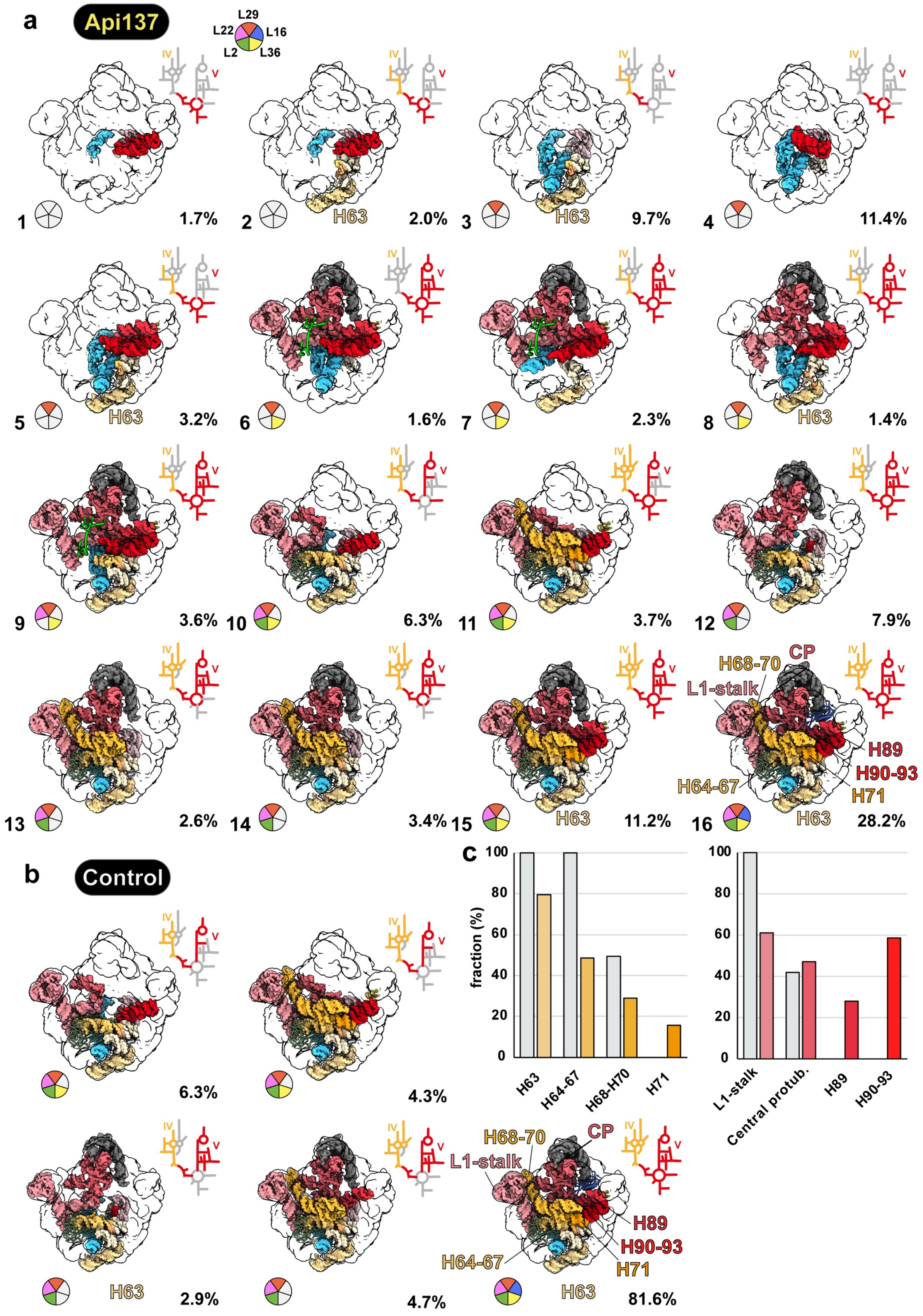

Supplementary Fig. 9 | Structures of pre-50S intermediates suggesting delayed and rerouted assembly pathways upon Api137 treatment. a Structural arrangements of identified pre-50S states 1 to 15 and 50S of the Api137-treated sample. Helix H34 and helices of domains IV and V are shown as

surface models when present in precursor states. Circles indicate the presence of the early-binding L-proteins uL22 and uL29, the intermediate-binding L-protein uL2, and the late-binding L-proteins uL16 and bL36. **b** Same as **a** but shown for control precursor states 1 to 4 and 50S. **c** Proportion of structured regions for domain IV (left) and domain V helices (right) among all precursors. Light gray: control, colored: Api137.

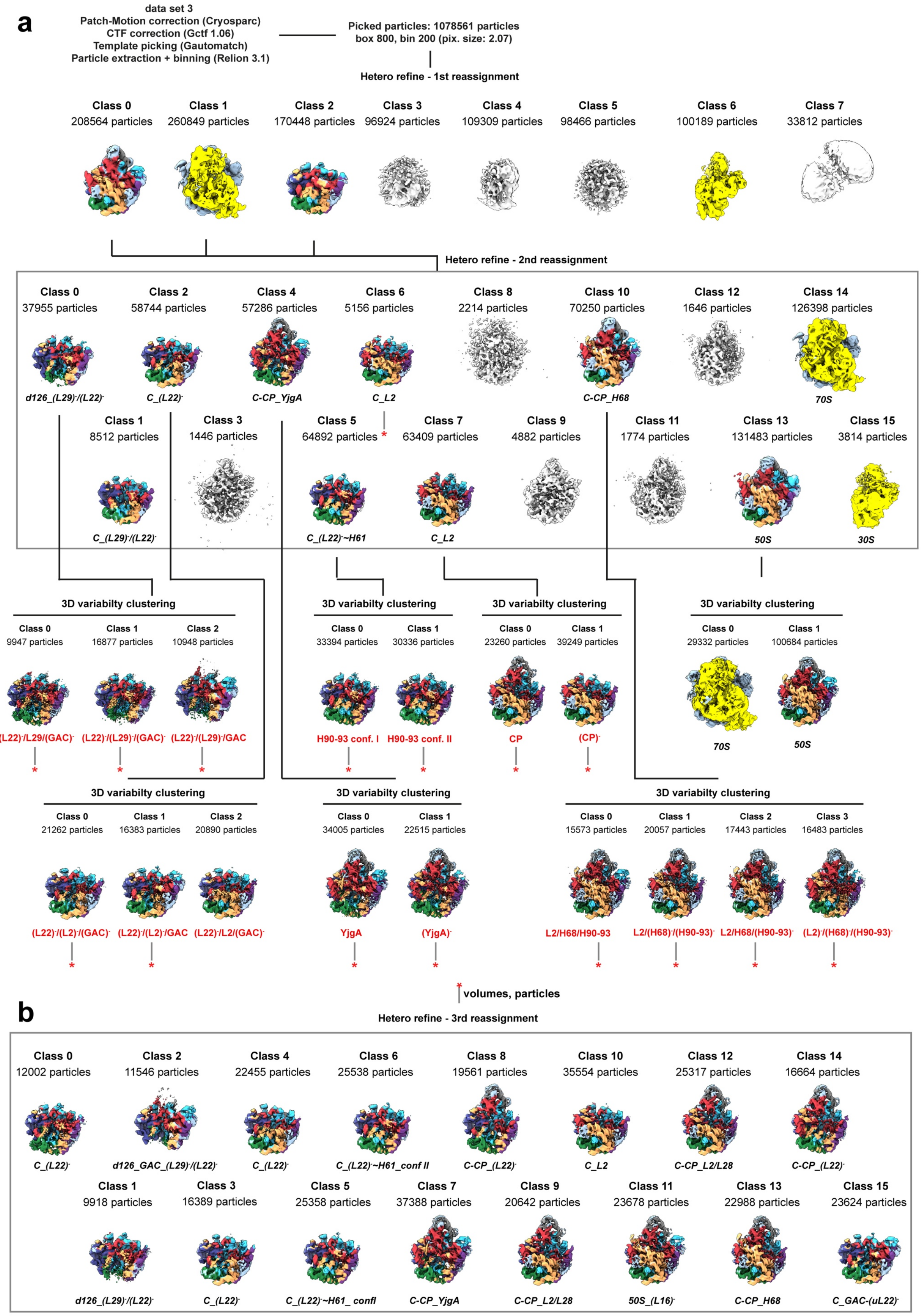
a
data set 3
Patch-Motion correction (Cryosparc)
CTF correction (Gctf 1.06)
Template picking (Gautomatch)
Particle extraction + binning (Relion 3.1)
Picked particles: 1078561 particles
box 800, bin 200 (pix. size: 2.07)
Hetero refine - 1st reassignment
Class 0 208564 particles
Class 1 260849 particles
Class 2 170448 particles
Class 3 96924 particles
Class 4 109309 particles
Class 5 98466 particles
Class 6 100189 particles
Class 7 33812 particles
Hetero refine - 2nd reassignment
Class 0 37955 particles d126_(L29)-/(L22)-
Class 2 58744 particles C_(L22)-
Class 4 57286 particles C-CP_YjgA
Class 6 5156 particles C_L2
Class 8 2214 particles
Class 10 70250 particles C-CP_H68
Class 12 1646 particles
Class 14 126398 particles 70S
Class 1 8512 particles C_(L29)-/(L22)-
Class 3 1446 particles
Class 5 64892 particles C_(L22)-~H61
Class 7 63409 particles C_L2
Class 9 4882 particles
Class 11 1774 particles
Class 13 131483 particles 50S
Class 15 3814 particles 30S
3D variabilty clustering
Class 0 9947 particles (L22)-/L29/(GAC)-
Class 1 16877 particles (L22)-/(L29)-/(GAC)-
Class 2 10948 particles (L22)-/(L29)-/GAC
3D variabilty clustering
Class 0 33394 particles H90-93 conf. I
Class 1 30336 particles H90-93 conf. II
3D variabilty clustering
Class 0 23260 particles CP
Class 1 39249 particles (CP)-
3D variabilty clustering
Class 0 29332 particles 70S
Class 1 100684 particles 50S
3D variabilty clustering
Class 0 21262 particles (L22)-/(L2)-/(GAC)-
Class 1 16383 particles (L22)-/(L2)-/GAC
Class 2 20890 particles (L22)-/L2/(GAC)-
3D variabilty clustering
Class 0 34005 particles YjgA
Class 1 22515 particles (YjgA)-
3D variabilty clustering
Class 0 15573 particles L2/H68/H90-93
Class 1 20057 particles L2/(H68)-/(H90-93)-
Class 2 17443 particles L2/H68/(H90-93)-
Class 3 16483 particles (L2)-/(H68)-/(H90-93)-
* volumes, particles
b
Hetero refine - 3rd reassignment
Class 0 12002 particles C_(L22)-
Class 2 11546 particles d126_GAC_(L29)-/(L22)-
Class 4 22455 particles C_(L22)-
Class 6 25538 particles C_(L22)-~H61_conf II
Class 8 19561 particles C-CP_(L22)-
Class 10 35554 particles C_L2
Class 12 25317 particles C-CP_L2/L28
Class 14 16664 particles C-CP_(L22)-
Class 1 9918 particles d126_(L29)-/(L22)-
Class 3 16389 particles C_(L22)-
Class 5 25358 particles C_(L22)-~H61_ confI
Class 7 37388 particles C-CP_YjgA
Class 9 20642 particles C-CP_L2/L28
Class 11 23678 particles 50S_(L16)-
Class 13 22988 particles C-CP_H68
Class 15 23624 particles C_GAC-(uL22)-

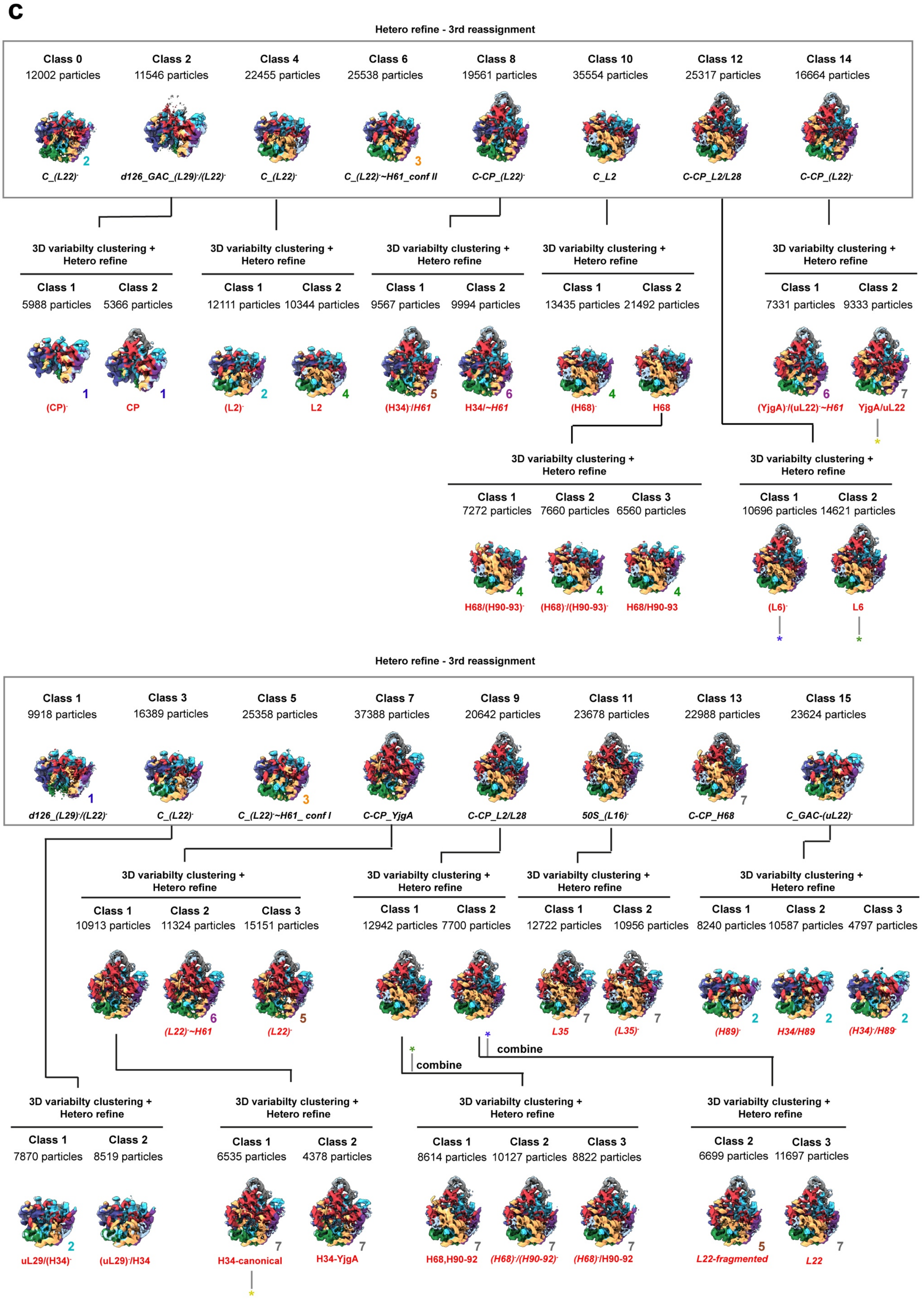
c
Hetero refine - 3rd reassignment
Class 0
12002 particles
C_(L22)-
Class 2
11546 particles
d126_GAC_(L29)-/(L22)-
Class 4
22455 particles
C_(L22)-
Class 6
25538 particles
C_(L22)-~H61_conf II
Class 8
19561 particles
C-CP_(L22)-
Class 10
35554 particles
C_L2
Class 12
25317 particles
C-CP_L2/L28
Class 14
16664 particles
C-CP_(L22)-
3D variabilty clustering +
Hetero refine
Class 1
5988 particles
(CP)-
Class 2
5366 particles
CP
3D variabilty clustering +
Hetero refine
Class 1
12111 particles
(L2)-
Class 2
10344 particles
L2
3D variabilty clustering +
Hetero refine
Class 1
9567 particles
(H34)-/H61
Class 2
9994 particles
H34/~H61
3D variabilty clustering +
Hetero refine
Class 1
13435 particles
(H68)-
Class 2
21492 particles
H68
3D variabilty clustering +
Hetero refine
Class 1
7331 particles
(YjgA)-/(uL22)-~H61
Class 2
9333 particles
YjgA/uL22
3D variabilty clustering +
Hetero refine
Class 1
7272 particles
H68/(H90-93)-
Class 2
7660 particles
(H68)-/(H90-93)-
Class 3
6560 particles
H68/H90-93
3D variabilty clustering +
Hetero refine
Class 1
10696 particles
(L6)-
Class 2
14621 particles
L6
Hetero refine - 3rd reassignment
Class 1
9918 particles
d126_(L29)-/(L22)-
Class 3
16389 particles
C_(L22)-
Class 5
25358 particles
C_(L22)-~H61_ conf I
Class 7
37388 particles
C-CP_YjgA
Class 9
20642 particles
C-CP_L2/L28
Class 11
23678 particles
50S_(L16)-
Class 13
22988 particles
C-CP_H68
Class 15
23624 particles
C_GAC-(uL22)-
3D variabilty clustering +
Hetero refine
Class 1
10913 particles
Class 2
11324 particles
(L22)-~H61
Class 3
15151 particles
(L22)-
3D variabilty clustering +
Hetero refine
Class 1
12942 particles
Class 2
7700 particles
3D variabilty clustering +
Hetero refine
Class 1
12722 particles
L35
Class 2
10956 particles
(L35)-
3D variabilty clustering +
Hetero refine
Class 1
8240 particles
(H89)-
Class 2
10587 particles
H34/H89
Class 3
4797 particles
(H34)-/H89-
combine
combine
3D variabilty clustering +
Hetero refine
Class 1
7870 particles
uL29/(H34)-
Class 2
8519 particles
(uL29)-/H34
3D variabilty clustering +
Hetero refine
Class 1
6535 particles
H34-canonical
Class 2
4378 particles
H34-YjgA
3D variabilty clustering +
Hetero refine
Class 1
8614 particles
H68,H90-92
Class 2
10127 particles
(H68)-/(H90-92)-
Class 3
8822 particles
(H68)-/H90-92
3D variabilty clustering +
Hetero refine
Class 2
6699 particles
L22-fragmented
Class 3
11697 particles
L22

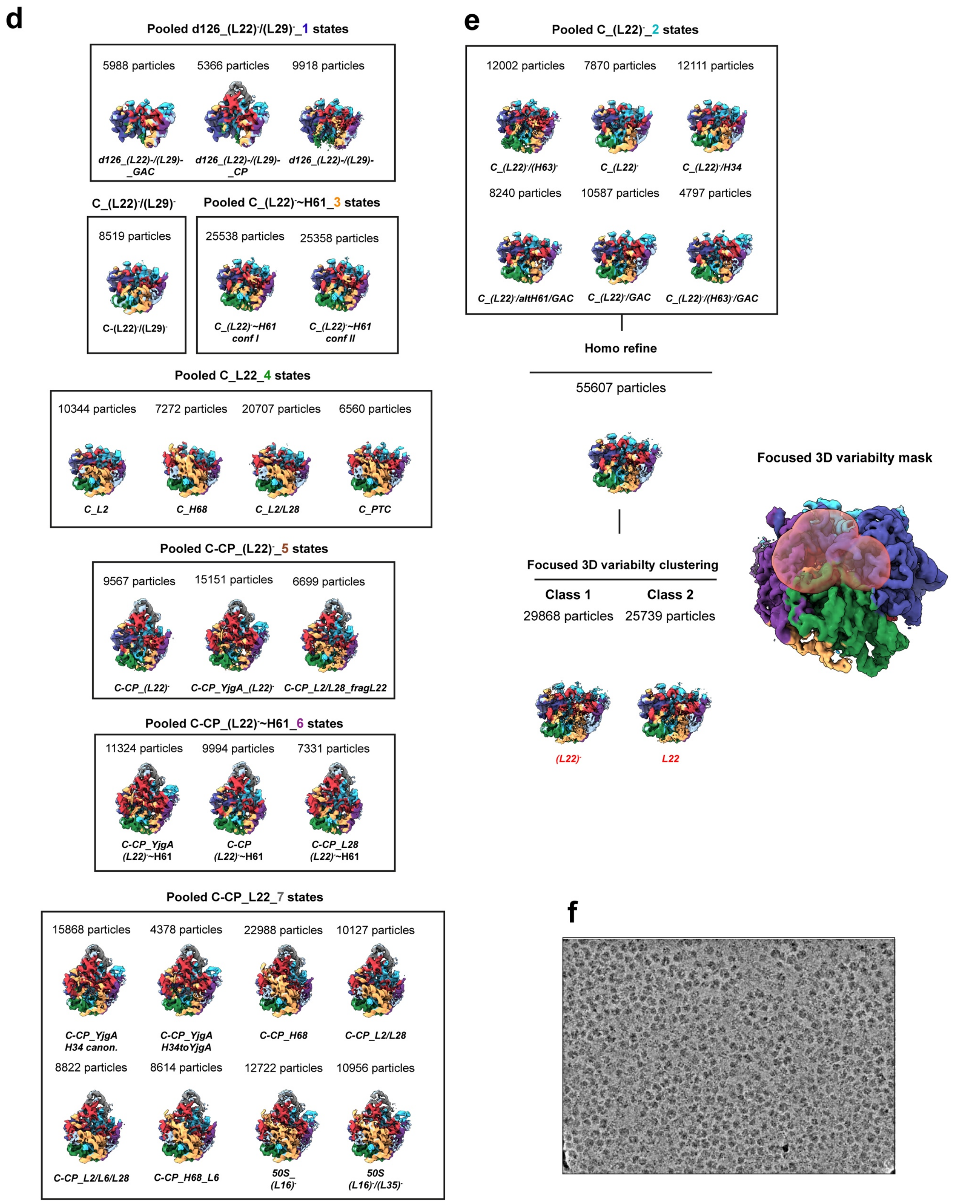

Supplementary Fig. 10 | Sorting scheme of the particles derived from Api137-treated cells supplemented with 30 µM Api137

a A third data set using purified precursors from Api137-treated cells with the addition of 30 µM Api137 was acquired. Again, generalized picking templates (see Methods) were used for template-based picking using Gautomatch. Particle images were extracted and normalized, using Relion 3.1 [45] with a box size of 800 and Fourier cropped to 200 for sorting. Newly extracted particles from this data set were reassigned to the previously identified 70S, 50S, 30S and pre-50S states and non-ribosomal classes (1st

reassignment). Next, particles from the 50S, 70S and pre-50S class were reassigned to previously identified states in the first Api137 data set (2nd reassignment). Multiple classes were non-informative and were discarded after inspection by a 2D classification. Ribosomal classes were subjected to one more round of 3D variability clustering. Red asterisks indicate classes that were used for the next reassignment **b** All ribosomal particles were re-assigned to the newly identified classes (3rd reassignment). **c** Further variability was sorted using hierarchical 3D variability clustering, resulting in 30 overall pre-50S classes. Green, yellow, and purple asterisks indicate highly similar subclasses that were combined. Colored numbers next to the cryo-EM maps, indicate classes with the same arrangement of Api137 density within the PET next to the exit site. **d, e** Final classes with the same compositions at the PET exit site were pooled. **e** Classes with fragmented or absent uL22 density were pooled and subjected to another round of focused 3D variability, resulting in a $(uL22)^-$ and a uL22 class. **f** Exemplary micrograph, low-pass filtered to 5 Å.

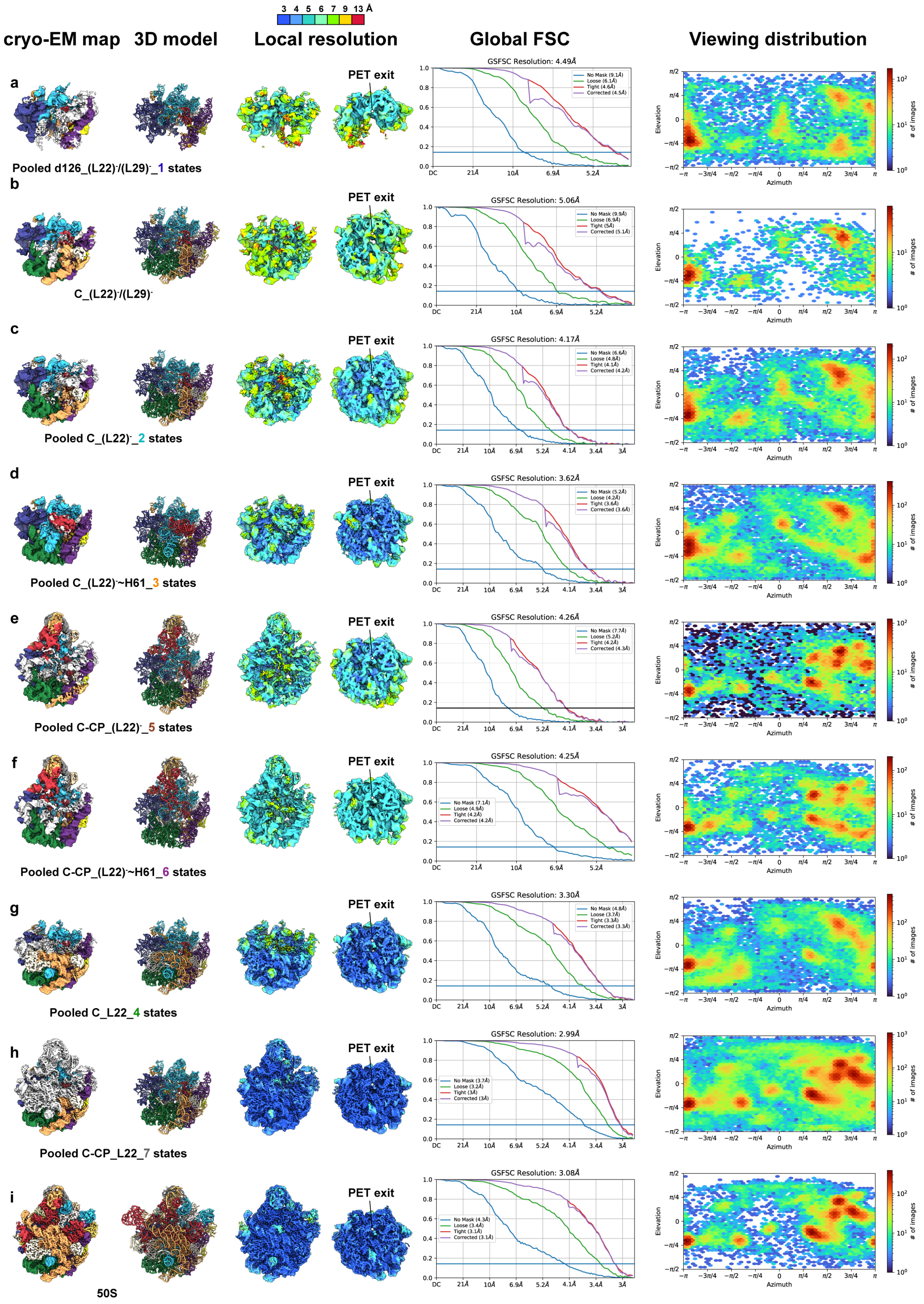

Supplementary Fig. 11 | cryo-EM maps, models, local and global resolutions and angular distributions of pooled refinements from pre-50S precursors in the presence of 30 µM Api137. a-i Cryo-EM maps of pooled refinements (see Suppl. Figure. 10) are shown. Invariant rRNA and L-protein

elements between individual sub-states within pooled classes are color-coded according to the six architectural domains of the 23S rRNA they are part of. Variant regions within these refinements are shown in white. PDB with invariant parts between sub-states are shown. For each state, the local resolution is shown in crown and bottom view. The Api137 PET exit bind site is indicated. Global FSC curves with estimated global resolutions at the 0.143 cut-off, as well as the angular distribution plots of the final reconstructions, are provided.

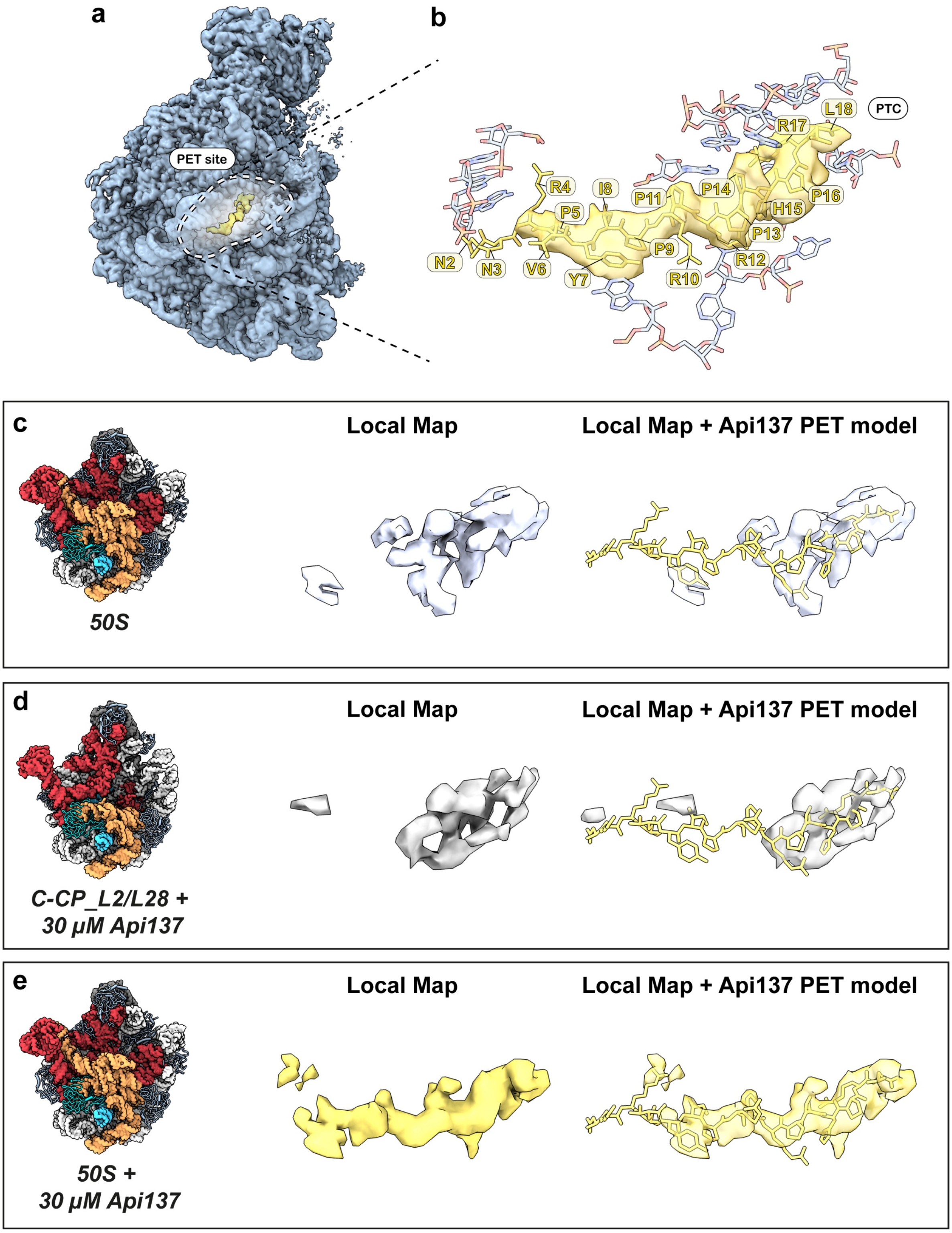

Supplementary Fig. 12 | Absence and presence of Api137 at the PET binding site

a Cryo-EM map of Api137 (yellow) bound to the 50S subunit (blue) at the PET binding site is shown. **b** Close-up of the Api137 binding site with the local density map of Api137 (transparent yellow) and the atomic model. Maps and models in **a** and **b** taken from Lauer et al., 2024 (PDB: 8RPY). **c-e** Local cryo-EM density maps for the PET binding site at a 4 Å distance from the atomic model of Api137 (rigid-body fit of PDB: 8RPY), with or without supplemented Api137. Local environments are shown

for **c** the 50S class derived from Api137-treated cells, **d** the 50S class derived from Api137-treated cells supplemented with 30 μM Api137, and **e** the late 50S assembly precursor *C-CP_L2/L28* derived from Api137-treated cells supplemented with 30 μM Api137. Maps were Gaussian filtered with a standard deviation of 1. Yellow-colored maps show the presence of Api137 at the PET binding site, and light grey when it is absent.

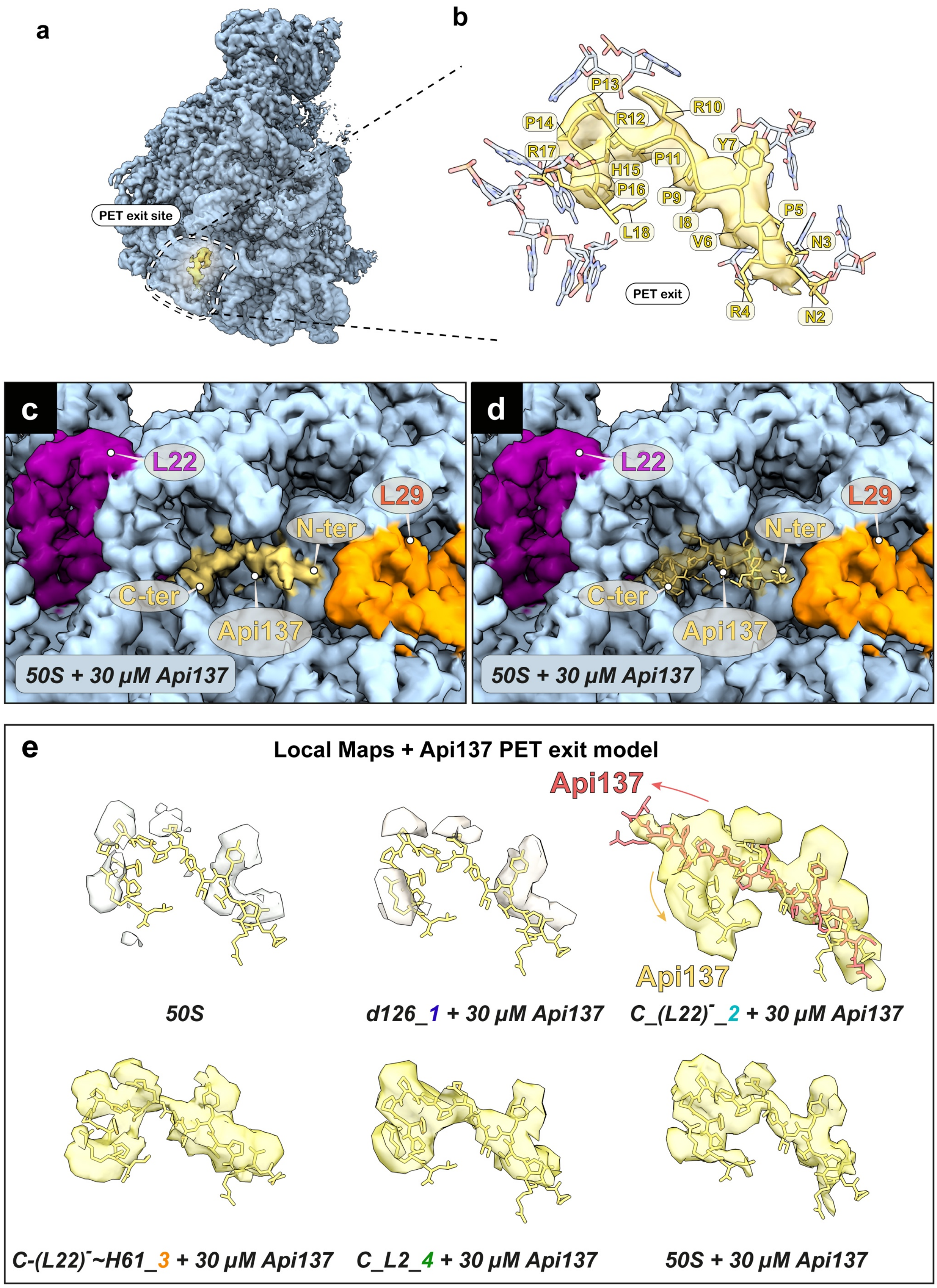

Supplementary Fig. 13 | Api137 binding to pre-50S precursors and 50S at the PET exit binding site

a Cryo-EM map of Api137 (yellow) bound to the 50S subunit (blue) at the PET exit binding site is shown. **b** Close-up of the Api137 PET exit binding site with the local density map of Api137 (transparent yellow) and the atomic model. Maps and models in **a** and **b** are taken from Lauer et al., 2024. **c, d** Cryo-EM density maps of the 50S supplemented with 30 µM Api137 are shown. uL22 (purple), uL29 (orange), and Api137 (yellow) at its PET exit site are colored. Api137 is shown either opaque (**c**) or transparent (**d**) with the atomic model rigid-body fitted. Atomic model (PDB: 8RPY) taken from Lauer et al., 2024. **e** Local density maps for the PET exit binding site of different pooled states at a 4 Å distance from the atomic model of Api137 (rigid-body fit of PDB: 8RPY), without or with supplementation of Api137. Colored numbers indicate pooled classes with the same organizations at the PET exit site (see Supplementary Figure 10d, e). Maps were Gaussian filtered with a standard deviation of 1. Light grey cryo-EM maps indicate background, yellow-colored maps represent Api137 at the PET exit binding site.

Supplementary Table 1: Cryo-EM data collection, refinement, and validation statistics for the control data set

	#1 *Ctrl_ C_L2*	#2 *Ctrl_ C_H68*	#3 *Ctrl_ C-CP_L2 (L22)$^{-}$*	#4 *Ctrl_ C-CP_H68 (L22)$^{-}$~H61*	#5 *Ctrl_ 50S*
**Data collection and processing**					
Magnification	81,000x	81,000x	81,000x	81,000x	81,000x
Voltage (kV)	300	300	300	300	300
Electron exposure (e–/Å^2)	45	45	45	45	45
Defocus range (μm)	-0.8-2.0	-0.8-2.0	-0.8-2.0	-0.8-2.0	-0.8-2.0
Pixel size (Å)	0.53 (2.12)	0.53 (2.12)	0.53 (2.12)	0.53 (2.12)	0.53 (2.12)
Symmetry imposed	C1	C1	C1	C1	C1
Initial particle images (no.)	752,255	752,255	752,255	752,255	752,255
Final particle images (no.)	10,627	7,221	4,800	7,937	136,185
Map resolution (Å)	4.35	4.35	4.39	4.84	4.35
FSC threshold	0.143	0.143	0.143	0.143	0.143
**Refinement**					
Initial model used (PDB code)	8C96	8C92	8C92	8C92	6GC8
Model resolution (Å)	4.3	4.3	4.3	4.6	4.3
FSC threshold	0.143	0.143	0.143	0.143	0.143
Model resolution range (Å)	4.3-5.7	4.3-7.3	4.2-6.7	4.6-7.6	4.3-4.4
Map sharpening *B* factor (Å^2)	-62.8	-40.3	-40.3	-54.2	-62.8
Model composition					
Non-hydrogen atoms	60,216	70,943	73,704	80,332	90,280
Protein residues	1715	2,194	2,330	2,664	3,265
RNA residues	2181	2,515	2,585	2,779	3,020
B factors (Å^2)					
Protein	-37.91	-45.36	-40,87	-138.35	-50.19
RNA	-38.84	-51.99	-64,3	-166.86	-52.14
R.m.s. deviations					
Bond lengths (Å)	0.002(0)	0.002(0)	0.005(1)	0.005(10)	0.002 (0)
Bond angles (°)	0.531(2)	0.552(4)	0.731(14)	0.551(12)	0.502(5)
Validation					
MolProbity score	1.92	1.83	1.89	2.06	1.92
Clashscore	9.25	8.56	8.31	8.48	11.14
Poor rotamers (%)	1.44	1.13	0	2.09	0
Ramachandran plot					
Favored (%)	95.53	95.36	93.24	94.86	94.89
Allowed (%)	4.35	4.55	6.68	5.14	4.99
Disallowed (%)	0.12	0.09	0.09	0	0.12

Supplementary Table 2: Cryo-EM data collection, refinement, and validation statistics for the data set from Api137-treated cells

	#1	#2	#3	#4	#5	#6	#7	#8
	d126 $(L29)^{-}$/ $(L22)^{-}$	*C* $(L29)^{-}$/ $(L22)^{-}$	*C* $(L22)^{-}$	*C* $(L22)^{-}$~H61	*C* *GAC* $(L22)^{-}$	*C-CP* $(L22)^{-}$	*C-CP* *YjgA* $(L22)^{-}$~H61	*C-CP* *YjgA* $(L22)^{-}$
	EMD 51828	EMD 51829	EMD 51830	EMD 51831	EMD 51832	EMD 51833	EMD 51834	EMD 51835
	PDB 9H3K	PDB 9H3L	PDB 9H3M	PDB 9H3N	PDB 9H3O	PDB 9H3P	PDB 9H3Q	PDB 93H3R
**Data collection and processing**								
Magnification	81,000x	81,000x	81,000x	81,000x	81,000x	81,000x	81,000x	81,000x
Voltage (kV)	300	300	300	300	300	300	300	300
Electron exposure (e–/Å^{2})	45	45	45	45	45	45	45	45
Defocus range (μm)	-0.8-2.0	-0.8-2.0	-0.8-2.0	-0.8-2.0	-0.8-2.0	-0.8-2.0	-0.8-2.0	-0.8-2.0
Pixel size (Å)	0.53 (1.41)	0.53 (1.41)	0.53 (1.41)	0.53 (1.41)	0.53 (1.41)	0.53 (2.12)	0.53 (1.41)	0.53 (1.41)
Symmetry imposed	C1	C1	C1	C1	C1	C1	C1	C1
Initial particle images (no.)	1,143,897	1,143,897	1,143,897	1,143,897	1,143,897	1,143,897	1,143,897	653,029
Final particle images (no.)	4,928	5,679	27,633	32,489	9,154	4,031	4,678	6,435
Map resolution (Å)	6.62	5.84	4.41	3.69	4.54	7.06	4.02	4.12
FSC threshold	0.143	0.143	0.143	0.143	0.143	0.143	0.143	0.143
Map resolution range (Å)	3.66-77	3.64-83	3.056-53	3.19-47	3.056-56	4.55-93	3.084-54	3.056-54
**Refinement**								
Initial model used (PDB code)	8C96	8C92	8C92	8C92	8C92 + 6GC8	8C90 + 6GC8	8C90 + 6GC8	8C90 + 6GC8
Model resolution (Å)	6.5	5.8	4.2	3.7	4.5	7.1	4.0	4.3
FSC threshold	0.143	0.143	0.143	0.143	0.143	0.143	0.143	0.143
Model resolution range (Å)	6.5-8.9	5.6-8.9	4.2-5.3	3.6-4.9	4.5-7.3	6.8-10.8	3.9-6.6	4.0-6.8
Map sharpening *B* factor (Å^{2})	-139.5	-98	-79	-58.9	-50.9	-91.8	-24.7	-36.1
Model composition								
Non-hydrogen atoms	28,210	53,578	53,135	52,447	61,407	74,661	75,644	77,864
Protein residues	1055	1524	1464	1306	1735	2487	2,291	2526
RNA residues	1399	1946	1941	1969	2231	2573	2689	2706
B factors (Å^{2})								
Protein	-184.13	-106.06	-108	-71.79	-100.78	-60.28	-73.85	-175.19
RNA	-233.70	-149.60	-156.98	-177.34	-157.10	-109.62	-63.63	354.94
R.m.s. deviations								
Bond lengths (Å)	0002 (0)	0.002(4)	0.002(0)	0.002(0)	0.006(8)	0.002(4)	0.002(0)	0.002(0)
Bond angles (°)	0.500 (0)	0.637 (7)	0.490(0)	0.473(5)	0.590(14)	0.629(7)	0.505(0)	0.531(7)
Validation								
MolProbity score	1.72	1.64	1.56	1.27	2.03	1.94	1.61	1.66
Clashscore	6.71	5.51	5.45	4.95	7.62	7.28	6.41	7.51
Poor rotamers (%)	0.36	0.18	0.34	0	1.78	1.24	0.38	0.54
Ramachandran plot								
Favored (%)	94,97	95.05	96.16	97.96	93.58	92.55	96.17	96,32
Allowed (%)	4.94	4.95	3.84	2.04	6.07	7.12	3.74	3,59
Disallowed (%)	0.1	0.00	0	0.08	0.35	0.33	0.09	0.08

	#9	#10	#11	#12	#13	#14	#15	#16
	C-CP	*C*	*C*	*C-CP*	*C-CP*	*C-CP*	*50S*	*50S*
	YjgA	*L2*	*L2-H68*	*L2-L28*	*L2-H68*	*L2-L35-H68*	*(L16)*$^-$	
	EMD 51836	EMD 51837	EMD 51838	EMD 51839	EMD 51840	EMD 51841	EMD 51842	EMD 51843
	PDB 9H3S	PDB 9H3T	PDB 9H3U	PDB 9H3V	PDB 9H3W	PDB 9H3X	PDB 9H3Y	PDB 9H3Z
**Data collection and processing**								
Magnification	81,000x	81,000x	81,000x	81,000x	81,000x	81,000x	81,000x	81,000x
Voltage (kV)	300	300	300	300	300	300	300	300
Electron exposure (e–/Å^2)	45	45	45	45	45	45	45	45
Defocus range (μm)	-0.8-2.0	-0.8-2.0	-0.8-2.0	-0.8-2.0	-0.8-2.0	-0.8-2.0	-0.8-2.0	-0.5-2.0
Pixel size (Å)	0.53 (2.12)	0.53 (2.12)	0.53 (2.12)	0.53 (2.12)	0.53 (2.12)	0.53 (2.12)	0.53 (2.12)	0.53 (2.12)
Symmetry imposed	C1	C1	C1	C1	C1	C1	C1	C1
Initial particle images (no.)	1,143,897	1,143,897	1,143,897	1,143,897	1,143,897	1,143,897	1,143,897	1,143,897
Final particle images (no.)	10,197	17,970	10,420	22,443	7,450	9,591	31,919	80,374
Map resolution (Å)	4.16	3.85	3.47	3.55	5.38	4.12	3.09	2.98
FSC threshold	0.143	0.143	0.143	0.143	0.143	0.143	0.143	0.143
Map resolution range (Å)	3.084-54	3.08-53	3.11-48	3.11-47	3.0-73	3.084-55	3.116-42	3.084-43
**Refinement**								
Initial model used (PDB code)	8C90 + 6GC8	8C91	8C91 + 6GC8	8C8Y	8C8X	6GC8	6GC8	6GC8
Model resolution (Å)	4.1	3.9	3.5	3.5	5.4	4.1	3.1	3.1
FSC threshold	0.143	0.143	0.143	0.143	0.143	0.143	0.143	0.143
Model resolution range (Å)	4.1-6.6	3.8-7.1	3.4-4.9	3.5-4.7	4.2-9.0	4.0-7.3	3.1-3.9	3.1-3.8
Map sharpening *B* factor (Å^2)	-37.2	-43.6	-25.3	-35.8	-71.7	-31.1	-36.6	-47.5
Model composition								
Non-hydrogen atoms	82,487	69,116	73,763	76,075	79715	80,393	88,357	89,947
Protein residues	2762	2,364	2,285	2,566	2594	2708	3,036	3,222
RNA residues	2835	2,361	2,607	2,609	2769	2758	3,015	3,020
B factors (Å^2)								
Protein	-67.48	-41.28	-43.14	-40.59	-137.68	-138.87	-49.58	-49.21
RNA	-51.32	-46.35	51.34	65.41	-166.72	-166.61	-51.89	-52.14
R.m.s. deviations								
Bond lengths (Å)	0.002 (0)	0.002(0	0.002(0	0.002(0)	0.004(10)	0.004(10)	0.002(0)	0.002(0)
Bond angles (°)	0.567 (6)	0.531 (0)	0.532(2)	0.525(1)	0.534(10)	0.537(10)	0.536(0)	0.546(2)
Validation								
MolProbity score	1.61	1.57	1.62	1.67	1.72	1.7	1.60	1.55
Clashscore	5.82	5.95	5.65	5.82	6.93	6.65	5.39	5.62
Poor rotamers (%)	0.53	0.68	0.7	0.48	0.38	0.45	0.65	0.88
Ramachandran plot								
Favored (%)	95.76	96.30	95.55	94.94	95.08	95.25	95.68	96.3
Allowed (%)	4.17	3.62	4.37	5.02	4.92	4.75	4.26	3.6
Disallowed (%)	0.07	0.09	0.09	0.04	0.00	0.00	0.07	0.09

Supplementary Table 3: Cryo-EM data collection, refinement, and validation statistics for the data set from Api137-treated cells supplemented with Api137

	#1	#2	#3	#4	#5	#6	#7
	Pooled d126_(L29)$^-$/ (L22)$^-$	*C (L29)$^-$/ (L22)$^-$*	*Pooled C (L22)$^-$ Canonical Api137*	*Pooled C (L22)$^-$* Alternative Api137	*Pooled C (L22)$^-$~H61*	*Pooled C-CP (L22)$^-$*	*Pooled C-CP (L22)$^-$~H61*
	EMD xxx PDB xxx	EMD xxx PDB xxx	EMD xxx PDB xxx	EMD xxx PDB xxx	EMD xxx PDB xxx	EMD xxx PDB xxx	EMD xxx PDB xxx
**Data collection and processing**							
Magnification	81,000x	81,000x	81,000x	81,000x	81,000x	81,000x	81,000x
Voltage (kV)	300	300	300	300	300	300	300
Electron exposure (e–/Å^2)	45	45	45	45	45	45	45
Defocus range (μm)	-0.8-2.0	-0.8-2.0	-0.8-2.0	-0.8-2.0	-0.8-2.0	-0.8-2.0	-0.8-2.0
Pixel size (Å)	0.666 (1.998)	0.666 (1.998)	0.666 (1.332)	0.666 (1.332)	0.666 (1.332)	0.666 (1.998)	0.666 (1.998)
Symmetry imposed	C1	C1	C1	C1	C1	C1	C1
Initial particle images (no.)	1,078,561	1,078,561	1,078,561	1,078,561	1,078,561	1,078,561	1,078,561
Final particle images (no.)	21,282	8,519	29,868	29,868	50,896	31,417	28,649
Map resolution (Å)	4.49	5.06	4.17	4.17	3.62	4.25	4.25
FSC threshold	0.143	0.143	0.143	0.143	0.143	0.143	0.143
**Refinement**							
Initial model used (this study)	*d126_(L29)$^-$/ (L22)$^-$*	*C (L29)$^-$/ (L22)$^-$*	*C (L22)$^-$*	*C (L22)$^-$*	*C (L22)$^-$~H61*	*C-CP (L22)$^-$*	*C-CP_YjgA_ (L22)$^-$~H61*
Model resolution (Å)	6.5	5.8	4.2	4.2	3.7	4.2	4.1
FSC threshold	0.143	0.143	0.143	0.143	0.143	0.143	0.143
Model resolution range (Å)	6.5-8.9	5.6-8.9	4.2-5.3	4.2-5.3	3.6-4.9	4.0-5.6	4.0-5.6
Map sharpening *B* factor (Å^2)	-47.7	-43.9	-70.2	-70.2	-66.1	-63.2	-62.4
Model composition							
Non-hydrogen atoms	36,717	53,578	50,932	50,932	52,595	60,234	59,416
Protein residues	1055	1524	1481	1481	1323	1940	1,807
RNA residues	1329	1946	1832	1832	1969	2096	2,108
B factors (Å^2)							
Protein	-184.13	-106.06	-107.27	-107.27	-71.49	-59.42	-48.91
RNA	-244.21	-149.60	-151.95	-151.95	-177.34	-100.94	-47.05
R.m.s. deviations							
Bond lengths (Å)	0003 (0)	0.002(4)	0.002(0)	0.002(0)	0.002(0)	0.002(0)	0.002(0)
Bond angles (°)	0.510 (0)	0.637 (7)	0.500(0)	0.502(1)	0.478(5)	0.507(3)	0.516(0)
Validation							
MolProbity score	1.75	1.64	1.75	1.77	1.4	2.05	1.83
Clashscore	7.28	5.51	8.86	9.08	6.04	8.68	10.15
Poor rotamers (%)	0.36	0.18	0.41	0.58	0.19	1.92	0.55
Ramachandran plot							
Favored (%)	94,97	95.05	95.99	95.85	97.96	94.78	95.65
Allowed (%)	4.94	4.95	4.01	4.08	2.04	5.16	4.24
Disallowed (%)	0.1	0.00	0	0.07	0.08	0.05	0.11

	#8	#9	#10
	Pooled	*Pooled*	
	C	*C-CP*	*50S*
	L2	*L2-L28*	
	EMD xxx	EMD xxx	EMD xxx
	PDB xxx	PDB xxx	PDB xxx
**Data collection and processing**			
Magnification	81,000x	81,000x	81,000x
Voltage (kV)	300	300	300
Electron exposure (e–/Å^2)	45	45	45
Defocus range (μm)	-0.8-2.0	-0.8-2.0	-0.5-2.0
Pixel size (Å)	0.666 (1.332)	0.666 (1.332)	0.666 (1.332)
Symmetry imposed	C1	C1	C1
Initial particle images (no.)	1,078,561	1,078,561	1,078,561
Final particle images (no.)	45,271	154,257	41,476
Map resolution (Å)	3.30	2.99	3.08
FSC threshold	0.143	0.143	0.143
**Refinement**			
Initial model used (this study)	*C* *L2*	*C-CP* *L2-L28*	*50S*
Model resolution (Å)	3.3	3.1	3.1
FSC threshold	0.143	0.143	0.143
Model resolution range (Å)	3.1-4.1	2.8-3.3	3.1-3.8
Map sharpening *B* factor (Å^2)	-46.6	-61.3	-41.8
Model composition			
Non-hydrogen atoms	60,486	73,870	90,242
Protein residues	2,076	1,754	3,256
RNA residues	2,061	1,869	3,020
B factors (Å^2)			
Protein	-38.05	-26.89	-49.21
RNA	-38.20	-45.12	-52.14
R.m.s. deviations			
Bond lengths (Å)	0.002(0)	0.003(0)	0.002(0)
Bond angles (°)	0.537 (0)	0.553(4)	0.546(2)
Validation			
MolProbity score	1.79	1.75	1.55
Clashscore	9.63	6.58	5.62
Poor rotamers (%)	0.77	0.49	0.88
Ramachandran plot			
Favored (%)	95.93	94.28	96.3
Allowed (%)	3.97	5.66	3.6
Disallowed (%)	0.1	0.06	0.09
