# Supplementary material for: The proline-rich antimicrobial peptide Api137 disrupts large ribosomal subunit assembly and induces misfolding
Source: Nat Commun. 2025 Jan 10;16:567. doi: 10.1038/s41467-025-55836-8 (PMC11723945; doi:10.1038/s41467-025-55836-8)
Supplement: Supplementary file 2 — Reporting Summary [file 41467_2025_55836_MOESM2_ESM.pdf]

nature portfolio

Corresponding author(s): Rainer Nikolay
Christian Spahn
Ralf Hoffmann

Last updated by author(s): Dec 15, 2024

Reporting Summary

Nature Portfolio wishes to improve the reproducibility of the work that we publish. This form provides structure for consistency and transparency in reporting. For further information on Nature Portfolio policies, see our Editorial Policies and the Editorial Policy Checklist.

Statistics

For all statistical analyses, confirm that the following items are present in the figure legend, table legend, main text, or Methods section.

n/a	Confirmed	
☐	☒	The exact sample size (*n*) for each experimental group/condition, given as a discrete number and unit of measurement
☐	☒	A statement on whether measurements were taken from distinct samples or whether the same sample was measured repeatedly
☐	☒	The statistical test(s) used AND whether they are one- or two-sided<br>*Only common tests should be described solely by name; describe more complex techniques in the Methods section.*
☒	☐	A description of all covariates tested
☒	☐	A description of any assumptions or corrections, such as tests of normality and adjustment for multiple comparisons
☐	☒	A full description of the statistical parameters including central tendency (e.g. means) or other basic estimates (e.g. regression coefficient) AND variation (e.g. standard deviation) or associated estimates of uncertainty (e.g. confidence intervals)
☐	☒	For null hypothesis testing, the test statistic (e.g. *F*, *t*, *r*) with confidence intervals, effect sizes, degrees of freedom and *P* value noted<br>*Give P values as exact values whenever suitable.*
☒	☐	For Bayesian analysis, information on the choice of priors and Markov chain Monte Carlo settings
☒	☐	For hierarchical and complex designs, identification of the appropriate level for tests and full reporting of outcomes
☒	☐	Estimates of effect sizes (e.g. Cohen's *d*, Pearson's *r*), indicating how they were calculated

Our web collection on statistics for biologists contains articles on many of the points above.

Software and code

Policy information about availability of computer code

Data collection	For data collection a Titan Krios G3i transmission electron microscope (Thermo Fisher Scientific, Server version 2.15.3, TIA version 5.0) equipped with a K3 direct electron detector (Gatan, Digital Micrograph version 3.32.2403.0) was used. Images were acquired using the EPU software version 2.8.1 (Thermo Fischer Scientific).
Data analysis	CryoSPARC was used for pre-processing micrographs, correcting CTFs, picking and extracting particles. CryoSPARC and Relion 3.1 were used for initial reconstructions, 3D classifications, final refinements and to calculate global and local resolution. Coot v0.9.6 was used for manual model building. Phenix 1.20 was used for model refinement , validation and statistics. Figures were generated using ChimeraX v1.5, Adobe Illustrator 2024 and GraphPad Prism10 v10.1.2.

For manuscripts utilizing custom algorithms or software that are central to the research but not yet described in published literature, software must be made available to editors and reviewers. We strongly encourage code deposition in a community repository (e.g. GitHub). See the Nature Portfolio guidelines for submitting code & software for further information.

Data

Policy information about availability of data

All manuscripts must include a data availability statement. This statement should provide the following information, where applicable:

- Accession codes, unique identifiers, or web links for publicly available datasets
- A description of any restrictions on data availability
- For clinical datasets or third party data, please ensure that the statement adheres to our policy

Cryo-EM density maps and atomic models of pre-50S precursors from Api137-treated cells generated in this study have been deposited in EMDB and PDB as follows:
EMD-51828 [https://www.ebi.ac.uk/emdb/EMD-51828],
9H3K [http://doi.org/10.2210/pdb6YNV/pdb] (d126_(L29)-/(L22)-),
EMD-51829 [https://www.ebi.ac.uk/emdb/EMD-51829],
9H3L [http://doi.org/10.2210/pdb9H3L/pdb] (C_(L29)-/(L22)-),
EMD-51830 [https://www.ebi.ac.uk/emdb/EMD-51830],
9H3M [http://doi.org/10.2210/pdb9H3M/pdb] (C_(L22)-),
EMD-51831 [https://www.ebi.ac.uk/emdb/EMD-51831],
9H3N [http://doi.org/10.2210/pdb9H3N/pdb] (C_(L22)-~H61),
EMD-51832 [https://www.ebi.ac.uk/emdb/EMD-51832],
9H3O [http://doi.org/10.2210/pdb9H3O/pdb] (C_GAC_(L22)-),
EMD-51833 [https://www.ebi.ac.uk/emdb/EMD-51833],
9H3P [http://doi.org/10.2210/pdb9H3P/pdb] (C-CP_(L22)-),
EMD-51834 [https://www.ebi.ac.uk/emdb/EMD-51834],
9H3Q [http://doi.org/10.2210/pdb9H3Q/pdb] (C_YjgA_(L22)-),
EMD-51835 [https://www.ebi.ac.uk/emdb/EMD-51835],
9H3R [http://doi.org/10.2210/pdb9H3R/pdb] (C_YjgA_(L22)-~H61)
EMD-51836 [https://www.ebi.ac.uk/emdb/EMD-51836],
9H3S [http://doi.org/10.2210/pdb9H3S/pdb] (C_YjgA)
EMD-51837 [https://www.ebi.ac.uk/emdb/EMD-51837],
9H3T [http://doi.org/10.2210/pdb9H3T/pdb] (C_L2)
EMD-51838 [https://www.ebi.ac.uk/emdb/EMD-51838],
9H3U [http://doi.org/10.2210/pdb9H3U/pdb] (C_L2/H68)
EMD-51839 [https://www.ebi.ac.uk/emdb/EMD-51839],
9H3V [http://doi.org/10.2210/pdb9H3V/pdb] (C-CP_L2/L28)
EMD-51840 [https://www.ebi.ac.uk/emdb/EMD-51840],
9H3W [http://doi.org/10.2210/pdb9H3W/pdb] (C-CP_L2-H68)
EMD-51841 [https://www.ebi.ac.uk/emdb/EMD-51841],
9H3X [http://doi.org/10.2210/pdb9H3X/pdb] (C-CP_L2/L35-H68)
EMD-51842 [https://www.ebi.ac.uk/emdb/EMD-51842],
9H3Y [http://doi.org/10.2210/pdb9H3Y/pdb] (50S_(L16)-)
EMD-51843 [https://www.ebi.ac.uk/emdb/EMD-51843],
9H3Z [http://doi.org/10.2210/pdb9H3Z/pdb] (50S)
Cryo-EM density maps and truncated atomic models with rigid-body fitted Api137 of pooled pre-50S states from Api137-treated cells supplemented with Api137 generated in this study have been deposited in EMDB and PDB as follows:
EMD-51982 [https://www.ebi.ac.uk/emdb/EMD-51982],
9HAL [http://doi.org/10.2210/pdb9HAL/pdb] (Pooled d126_(L29)-/(L22)-_1);
EMD-51983[https://www.ebi.ac.uk/emdb/EMD-51983],
9HAM [http://doi.org/10.2210/pdb9HAM/pdb]] (C_(L29)-/(L22)-);
EMD-51973 [https://www.ebi.ac.uk/emdb/EMD-51973],
9HA1 [http://doi.org/10.2210/pdb9HA1/pdb] (Pooled C_(L22)-_2 with the canonical PET exit Api137 conformation);
EMD-51974 [https://www.ebi.ac.uk/emdb/EMD-51974],
9HA2 [http://doi.org/10.2210/pdb9HA2/pdb] (Pooled C_(L22)-_2 with the alternative PET exit Api137 conformation);
EMD-51975 [https://www.ebi.ac.uk/emdb/EMD-51975],
9HA3 [http://doi.org/10.2210/pdb9HA3/pdb] (Pooled C_(L22)-~H61_3);
EMD-51976 [https://www.ebi.ac.uk/emdb/EMD-51976],
9HA4 [http://doi.org/10.2210/pdb9HA4/pdb] (Pooled C-CP_(L22)-_5);
EMD-51979 [https://www.ebi.ac.uk/emdb/EMD-51979],
9HA7 [http://doi.org/10.2210/pdb9HA7/pdb] (Pooled C-CP_(L22)-~H61_6);
EMD-51977 [https://www.ebi.ac.uk/emdb/EMD-51977],
9HA5 [http://doi.org/10.2210/pdb9HA5/pdb] (Pooled C_L2_4);
EMD-51981[https://www.ebi.ac.uk/emdb/EMD-51981],
9HAI [http://doi.org/10.2210/pdb9HAI/pdb] (Pooled C_CP_7);
EMD-51978 [https://www.ebi.ac.uk/emdb/EMD-51978],
9HA6 [http://doi.org/10.2210/pdb9HA6/pdb] (50S);
The following cryo-EM density maps are available via Zenodo.
Control data set: zenodo entry 13939462 [https://doi.org/10.5281/zenodo. 13939462]
Individual pre-50S states from the Api137 treated sample supplemented with Api137:

zenodo entry 13919082 [https://doi.org/10.5281/zenodo.13919082]

We mandate deposition of all protein mass spectrometry raw data in a ProteomeXchange partner repository [https://panoramaweb.org/Api137_immature_ribo.url]. Source data are provided with this paper.

Research involving human participants, their data, or biological material

Policy information about studies with human participants or human data. See also policy information about sex, gender (identity/presentation), and sexual orientation and race, ethnicity and racism.

Reporting on sex and gender	N/A
Reporting on race, ethnicity, or other socially relevant groupings	N/A
Population characteristics	N/A
Recruitment	N/A
Ethics oversight	N/A

Note that full information on the approval of the study protocol must also be provided in the manuscript.

Field-specific reporting

Please select the one below that is the best fit for your research. If you are not sure, read the appropriate sections before making your selection.

☒ Life sciences ☐ Behavioural & social sciences ☐ Ecological, evolutionary & environmental sciences

For a reference copy of the document with all sections, see nature.com/documents/nr-reporting-summary-flat.pdf

Life sciences study design

All studies must disclose on these points even when the disclosure is negative.

Sample size	Sample sizes and number of replicates were based on successful experiments from previous publications. For each data set, cryo-EM micrographs were collected until more than 750,000 particles were obtained, ensuring sufficient data for comprehensive sorting.
Data exclusions	Micrographs with low estimated resolution or poorly fitted CTFs were discarded, along with particles that clustered into poorly defined classes during 3D classification.
Replication	To compare the growth of different E. coli strains under the influence of PrAMPs, at least three different biological replicates where measured. For the ribosome profile analysis of the fluorescently labeled strain, at least 6 different biological replicates were used due to the increased complexity of the process and the involvement of additional techniques and measuring devices. To ensure robust statistical analysis, 10 biological replicates were included, representing an optimal balance between effort and data reliability.
Randomization	Not relevant, as characterized compounds were individually tested and result independent of the investigator`s prior knowledge or hypothesis.
Blinding	Not relevant, as the results are independent of the investigator`s prior knowledge or hypothesis.

Reporting for specific materials, systems and methods

We require information from authors about some types of materials, experimental systems and methods used in many studies. Here, indicate whether each material, system or method listed is relevant to your study. If you are not sure if a list item applies to your research, read the appropriate section before selecting a response.

Materials & experimental systems

n/a	Involved in the study
☒	☐ Antibodies
☒	☐ Eukaryotic cell lines
☒	☐ Palaeontology and archaeology
☒	☐ Animals and other organisms
☒	☐ Clinical data
☒	☐ Dual use research of concern
☒	☐ Plants

Methods

n/a	Involved in the study
☒	☐ ChIP-seq
☒	☐ Flow cytometry
☒	☐ MRI-based neuroimaging
